# Omnipresent Maxwell's demons orchestrate information management in living cells

**DOI:** 10.1111/1751-7915.13378

**Published:** 2019-02-25

**Authors:** Grégory Boël, Olivier Danot, Victor de Lorenzo, Antoine Danchin

**Affiliations:** ^1^ UMR 8261 CNRS‐University Paris Diderot Institut de Biologie Physico‐Chimique 13 rue Pierre et Marie Curie 75005 Paris France; ^2^ Institut Pasteur 25‐28 rue du Docteur Roux 75724 Paris Cedex 15 France; ^3^ Molecular Environmental Microbiology Laboratory Systems Biology Programme Centro Nacional de Biotecnologia C/Darwin n° 3, Campus de Cantoblanco 28049 Madrid España; ^4^ Institute of Cardiometabolism and Nutrition Hôpital de la Pitié‐Salpêtrière 47 Boulevard de l'Hôpital 75013 Paris France; ^5^ The School of Biomedical Sciences Li Kashing Faculty of Medicine Hong Kong University 21, Sassoon Road Pokfulam SAR Hong Kong

## Abstract

The development of synthetic biology calls for accurate understanding of the critical functions that allow construction and operation of a living cell. Besides coding for ubiquitous structures, minimal genomes encode a wealth of functions that dissipate energy in an unanticipated way. Analysis of these functions shows that they are meant to manage information under conditions when discrimination of substrates in a noisy background is preferred over a simple recognition process. We show here that many of these functions, including transporters and the ribosome construction machinery, behave as would behave a material implementation of the information‐managing agent theorized by Maxwell almost 150 years ago and commonly known as Maxwell's demon (MxD). A core gene set encoding these functions belongs to the minimal genome required to allow the construction of an autonomous cell. These MxDs allow the cell to perform computations in an energy‐efficient way that is vastly better than our contemporary computers.

## Introduction

The ambition of synthetic biology to create a cell from first principles requires identification of the clusters of functions that allow the basic biological unit to be alive. Among other important achievements, genome annotation characterized the genes that direct the construction and operation of a functional cell. Many of these genes, for example those that drive the making of the ribosome and rule their behaviour, have a straightforward function. By contrast, we keep discovering new genes coding for unexpected functions pertaining to the operating system of the cell. We infer their activity as if it were the sequel of previous knowledge. In this context, we often face an omnipresent challenge, that of understanding the role of proteins spending energy apparently in a superfluous way, or proteins that dissipate energy while their obvious function does not entail such waste – e.g. why would a protease require ATP hydrolysis when its action is obviously exothermic? Why do many transporters hydrolyse two ATP molecules rather than only one? A careful analysis of the role of such proteins suggests that we deal, in all these cases, with the challenge of accurate recognition, when, to achieve its function, the protein must not only recognize a correct interaction but discriminate between similar ones and exclude those that are irrelevant. In engineered systems – typically built with hard materials – this is brought about by physically separating the components or endowing them with specific shapes and mechanical properties. But how is this achieved in the milieu of a cell, where so many events coincide in space and time operated by diffusible molecules and soft actors?

In life, to belong or not to belong is decisive. Making out this quandary is critical to understand how life proceeds. It is also critical to build up novel synthetic biology constructs that are able to scale up their designed functions. Here, after a preamble meant to clarify some deep properties of information usually less familiar to the life sciences scholar, we detail how discrimination can be achieved with a high level of accuracy by a variety of key proteins that behave as Maxwell's demons, microscopic agents invented by James Clerk Maxwell at the end of the XIXth century [MxDs, (Maxwell, 1871, reprinted 1902)]. Furthermore, we argue that many of these functions are essential to define what life is and must be encoded in the basic chassis of a living cell, a critical requirement for synthetic biology.

## Information in biology: history to the rescue

A widely accepted view of thermodynamics is that a specific energy cost is associated with the manipulation of information. Alas, how this materializes has not yet permeated the standard understanding of physics in its biological embodiment. It is still usual to think that creation of information requires energy (Szilard, 1929; von Neumann, 1966), but the way this pervasive (but wrong) idea is implemented in biological systems is widely ignored. To be sure, it is commonplace to read that genes and proteins are information‐loaded, but for us life scientists, this view does not entail consuming energy to achieve this very purpose. Yet, in the domain of physics, experiments have already transmuted information into work (Toyabe and Muneyuki, 2013; Masuyama *et al*., 2018), and this is at a time when biologists seldom take into quantitative consideration the physical reality of this authentic currency of nature (Landauer, 1996). It seems therefore worthwhile to outline here the way information is seen now, before exploring how it is brought into play in cells and encoded in their genome.

### Making information a physical currency

The way information is handled in biological systems is usually limited to fairly vague descriptions. Besides an ambiguous use of the word, most of the reality covered by the concept in quantitative terms is restricted to an analysis of the sequence of the building blocks that make the so‐called ‘informational macromolecules’. A classical illustration is provided by a coarse measure of a macromolecule's informational content, such as its ‘logo’, initially designed to characterize gene sequences – but not their biological meaning, then rapidly extended to proteins (Schneider and Stephens, 1990; Danchin, 1996; Schneider, 1997). Other places where information is important, such as the cell's organization, in particular its compartmentalization – essential to cope with chemical constraints (de Lorenzo *et al*., 2014), is seldom considered as requiring some type of quantification. All the same, information is a key descriptor of reality, and the laws that preside over its creation, destruction or propagation must be understood: after all, evolution of living organisms keeps creating novel information. As a genuine category of physics, information is associated with energy dissipation, governing the dynamics of physical, chemical and biological systems. If information is a concrete feature of living matter, there must exist genes that deal with it, beyond their informational description. Even more significant, these genes should be essential to understand what life is. This would imply that understanding what they are and how they fare is critical when studying genes and genomes while planning to use them in synthetic biology constructs.

How could we access them? A short history of the concept will help us try and retrieve information‐specific genes, while carefully browsing genome annotations (Danchin *et al*., 2018). Back in 1961, Rolf Landauer established that, contrary to popular belief, computation is reversible. This had the unexpected, and, to many investigators, preposterous consequence that *creation of information does not consume energy* (Landauer, 1961). Energy was involved, however, but not where it was expected. Its dissipation was essential, not to create information but to reset the process that created the information of interest. Briefly, erasing the memory of past events is energy‐costly. To make things clear, Charles Bennett illustrated reversible computation by showing how to build up a simple arithmetic operation, division, using the action of a MxD (Bennett, 1988a). In this process, Bennett emphasized the association between information and some ‘added value’ (more or less equating to the meaning of the information of interest) while pointing out the role of information in biology. The outcome of a division is obtained when a demon erases the intermediary states, leaving the rest of the division as the prominent ‘valuable’ outcome of the computation. In this process, memory erasure consumes energy. ‘The measurement itself can be performed reversibly, but an unavoidable entropy increase, which prevents the demon from violating the second law, occurs when the demon erases the result of one measurement to make room for the next’. The idea was then to copy the whole process that had led to the creation of the output and then erase the original file. All the energy involved was used in the erasing process, not in computing.

Yet, this view left open the need to erase only the part of the reversible computation that had produced the correct output. To identify the exact result of the procedure, we further need to use complementary (contextual) information that tells us where to look for the information just created, putting aside the information that was used to run the procedure reversibly. Briefly, the MxD must discriminate between what is significant and what is not. This remarkable feature results from the fact that the demon is not a random eraser but the result of natural selection, the process that associates information imbedded in genomes with contextual information. This very fact endowed MxDs with a goal (i.e. ability to take context into account for deciding): only those that behaved as if goal‐oriented survived and could belong to a live progeny of the cell [(Danchin and Sekowska, 2008; Danchin, 2009b) and see (van Hateren, 2015)]. Energy is used in living organisms to erase the overall memory of past actions *while preventing erasure of the goal of the operation* (the rest of the division in Bennett's illustration), a feature that results from natural selection. This results in a ratchet‐like discriminative process allowing information to accumulate. Under this frame, information capture would be a strong evolutionary drive, as any system with fluctuating components would tend to keep those energy‐neutral events that create information while discouraging those that erase it. Biological systems could thus spontaneously move towards information‐rich hubs in a functional solution space. Furthermore, as the information contents of a system increases, an evolutionary niche for emergence of information‐management devices can be reasonably anticipated. How could they look like? By dissipating energy, the residual memory is reset to a ground state that can be used for further computation, while keeping the result of the computation intact. In biology, this implies that we must identify concrete mechanisms that tell us what should be retained and what should be erased in the course of a particular biochemical pathway or other biological processes. To this end, we must identify processes that would comprise devices behaving as the microscopic agents invented by Maxwell.

**Figure 1:**
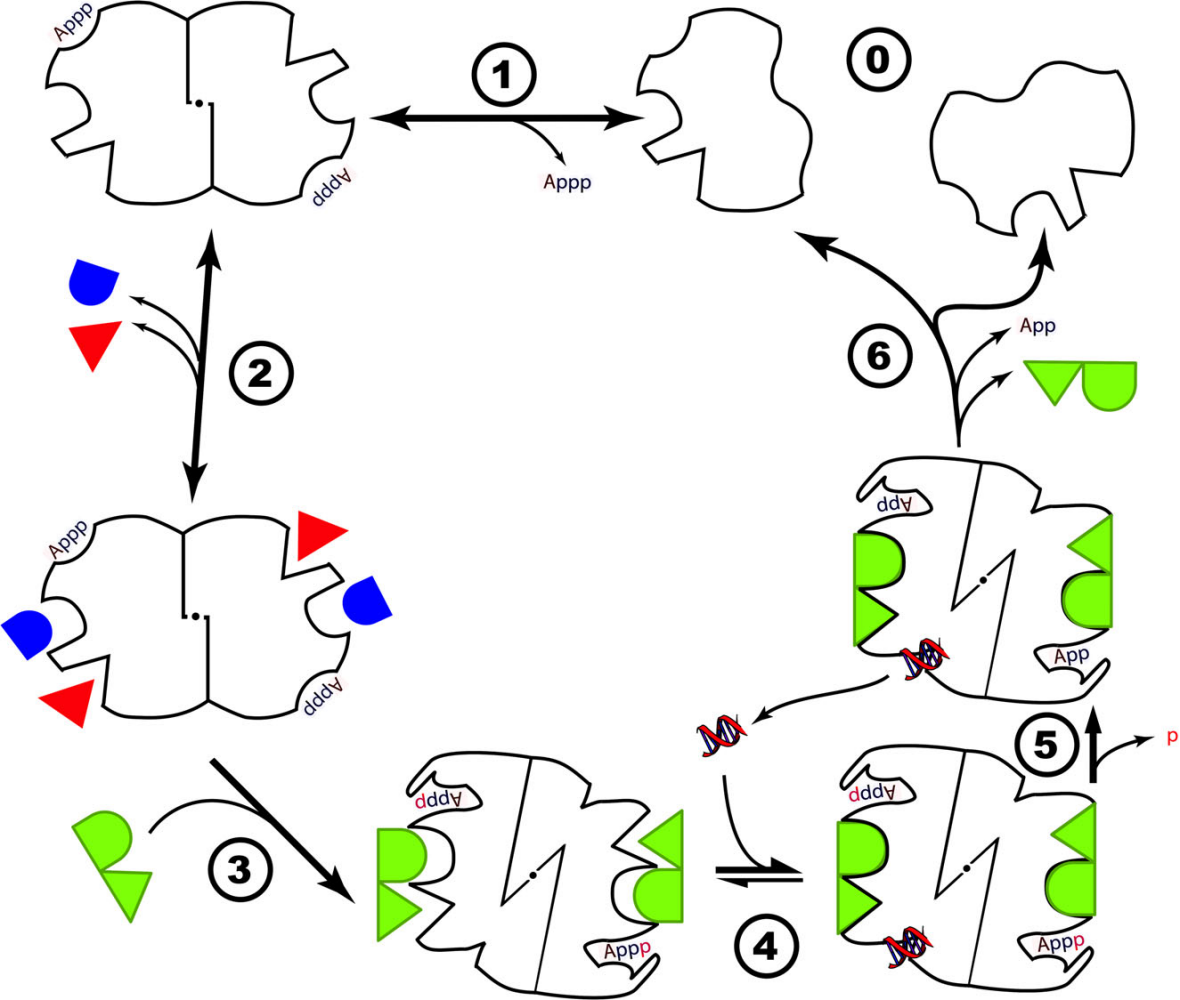
A discriminative molecular machine following Landauer's principle. We illustrate a molecular machine made of two subunits (stage 0). Upon weak binding with ATP, the subunits associate in a relaxed reversible manner (stage 1). The dimer can reversibly bind a variety of variants of the distinctive substrate (stage 2). Upon contact with the distinctive substrate, the dimer shifts to a tense form where the ATP hydrolysis energy barrier is lowered (stage 3). In this tense form, the dimer is able to recognize its specific target (stage 4, here illustrated as a fragment of nucleic acid) and this triggers irreversible ATP hydrolysis, freeing the target (stage 5). Subsequently (stage 6), ADP is released and the complex is reset to its original relaxed state. A variant of this scheme accounts for MalT regulation: there ADP remains bound during the first stages and is replaced by ATP upon binding of the correct substrate, maltotriose (see text).

Here, we review first how discrimination proceeds, then describe the general properties expected for a MxD [(Maxwell, 1871, reprinted 1902), and see how these views have been revived recently in the context of synthetic biology, e.g. (Danchin, 2009c; Mehta *et al*., 2016)]. We subsequently propose an open‐ended list of biological MxD functions likely to be implemented within cells. The list begins with discriminatory transport systems, which can be seen as downright embodiments of Maxwell's metaphor. Typically, creation of information involves a step where energy is loaded into a reversible ‘tense’ state of the protein of interest (e.g. a non‐hydrolysable analogue of ATP could substitute for ATP at this stage, and the process may involve multimerization of the protein), while a ratchet process, involving energy dissipation (e.g. ATP hydrolysis), makes the capture of information irreversible, while resetting the entity to its original ‘relaxed’ state [using the convenient nomenclature of the original model of allostery (Monod *et al*., 1965)], ready to start a new cycle again (Fig. 1).

### Complementarity in biology: discrimination versus recognition

Cells comprise thousands of specific entities with a large variation in size. Proper functioning requires that these entities interact with a variety of partners, often similar to one another, in a very accurate and faithful way. How is this performed? Among the physical laws that govern biology, complementarity plays a key role. An input of this concept from quantum physics was recognized as early as 1935 by Pascual Jordan, based on the idea of interaction between identical entities. This view, which is key to understand the emphasis still placed by many investigators on ‘self‐organisation’, was subsequently objected to and discussed by Linus Pauling and Max Delbrück, who, in 1940, created the modern concept of complementarity in biology. They showed that, to be valid, Jordan's view should be modified along lines that would entail taking into account the three dimensional structures of biological entities, usually not identical, but different from one another:

The novelty in Jordan's work lies in his suggestion that the well‐known quantum‐mechanical resonance phenomenon would lead to attraction between molecules containing identical groups […] It is our opinion that the processes of synthesis and folding of highly complex molecules in the living cell involve, in addition to covalent‐bond formation, only the intermolecular interactions of van der Waals attraction and repulsion, electrostatic interactions, hydrogen‐bond formation, etc., which are now rather well understood. These interactions are such as to give stability to a system of two molecules with complementary [emphasised by the authors] structures in juxtaposition, rather than of two molecules with necessarily identical structures; we accordingly feel that complementariness should be given primary consideration in the discussion of the specific attraction between molecules and the enzymatic synthesis of molecules. […] In order to achieve the maximum stability, the two molecules must have complementary surfaces, like die and coin, and also a complementary distribution of active groups (Pauling and Delbruck, 1940).

Beside foretelling complementarity in DNA thirteen years later, this description was remarkably befitting the well‐known hypothesis that Emil Fisher proposed in 1894 to describe a relationship between two biochemical structures, following the lock‐and‐key principle: ‘To use a metaphor, I would say that enzyme and substrate must fit together like lock and key in order to exert a chemical effect on each other. In any case, this notion becomes more likely and its value for stereochemical research increases when the phenomenon itself is transferred from the biological to the chemical realm’ [translated and cited in (Cramer, 1995)]. We should note here the caveat introduced by Fisher, suggesting that this metaphor might be less appropriate in biology than in standard chemistry. Indeed, recognition alone operates well in the absence of noise, such as competing structures, i.e. when the entity of interest must not only recognize its substrate but also discriminate against similar ones. This is the basis of the need for a special handling of structural information (directly or indirectly) by living cells, as we develop below.

Since the time of Emil Fisher, recognition between biological objects has been assumed to derive from key/lock recognition processes, but its demands in terms of thermodynamic requirements have seldom been explored in‐depth. To be sure, the metaphor is vividly illustrated by the way pharmaceutical companies still look out for drug targets, seeing drugs as jamming the lock or mimicking the proper substrate to make it work, but forgetting that, in addition to recognition, discrimination against similar targets is essential (this is the cause of most phase III clinical assays failures). Within cells, this matches the idea of generally free diffusion (except across cell membranes), where key‐like substrates explore their environment until they reach the proper lock, often an enzyme. During this process, information is measured as the substrate(drug)–target interaction, with little regard for competing entities. The key/lock metaphor extended to many other biological processes. For example, besides enzymes and their substrates, another common illustration was that of the recognition process allowing control of gene expression via interaction of transcription factors with regulatory molecules and their operator site or proper spatial localization of the object of interest.

Yet, molecular locks and keys are certainly not sufficiently accurate to ensure perfect order at the temperature of liquid water on Earth, where thermal energy is of the order of *kT*. This value is of the same order of magnitude as that of many hydrogen bonds, creating a considerable background noise. Key/lock recognition systems are too simple to afford the accuracy of 1/10 000 or even greater required in the synthesis of proteins and nucleic acids for example. This physics‐based challenge has been explored experimentally very early on. For example, Cramer and co‐workers illustrated the problem by comparing the input of valine and isoleucine analogues in the corresponding tRNA synthetases. They found that several energy‐consuming processes were implemented during the apparently simple process of ligation of an amino acid to its cognate tRNA (Englisch‐Peters *et al*., 1990). The conclusion was that standard recognition was far too low to allow accurate discrimination. This is especially true when amino acid analogues such as norvaline are found as inevitable side products of standard metabolism (Cvetesic *et al*., 2014). The amino acid must be checked for accuracy not only once but several times. The question of the initial selection and those that follow are fundamentally different, however. Beside key/lock recognition, further physical processes based on information management must be enforced. This is where the thought experiment involving a measuring widget, proposed 150 years ago by Maxwell, comes in.

### Maxwell's demon

**Figure 2:**
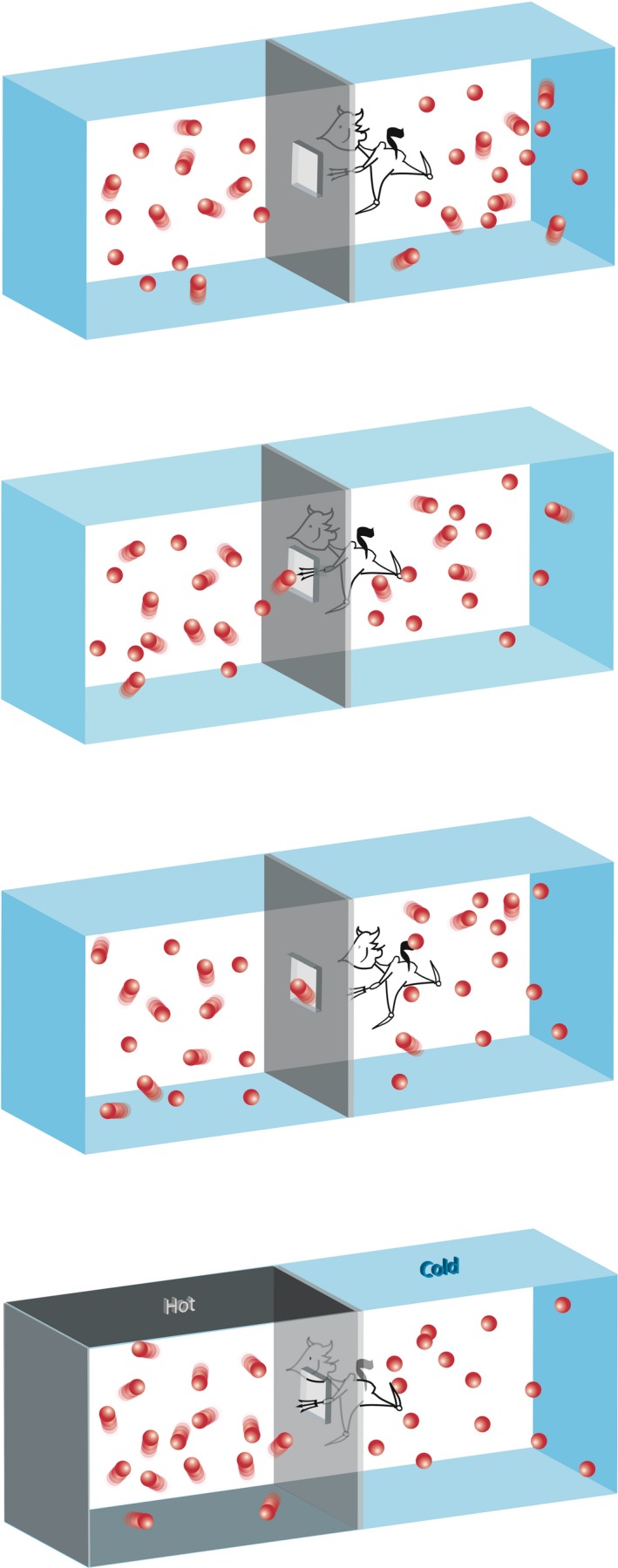
The original Maxwell's demon. The molecules in a vessel full of gas at uniform temperature are moving with highly variable velocities, though the mean velocity of any great number of them, arbitrarily selected, is almost exactly uniform. Now, let us suppose that such a vessel is divided into two compartments, left and right, by a division in which there is a small hole, and that a demon, who can see the individual molecules, opens and closes this hole, so as to allow only the swifter molecules to pass from right to left, and only the slower ones to pass from left to right. It will thus, without expenditure of work, raise the temperature of the left compartment and lower that of the right compartment, in apparent contradiction to the second law of thermodynamics.

Nothing is probably better than to use the very words Maxwell used to describe his demon (Fig. 2):

One of the best established facts in thermodynamics is that it is impossible in a system enclosed in an envelope which permits neither change of volume nor passage of heat, and in which both the temperature and the pressure are everywhere the same, to produce any inequality of temperature or of pressure without the expenditure of work. This is the second law of thermodynamics, and it is undoubtedly true as long as we can deal with bodies only in mass, and have no power of perceiving or handling the separate molecules of which they are made up. But if we conceive a being whose faculties are so sharpened that he can follow every molecule in its course, such a being, whose attributes are still as essentially finite as our own, would be able to do what is at present impossible to us. For we have seen that the molecules in a vessel full of air at uniform temperature are moving with velocities by no means uniform, though the mean velocity of any great number of them, arbitrarily selected, is almost exactly uniform. Now let us suppose that such a vessel is divided into two portions, A and B, by a division in which there is a small hole, and that a being, who can see the individual molecules, opens and closes this hole, so as to allow only the swifter molecules to pass from A to B, and only the slower ones to pass from B to A. He will thus, without expenditure of work, raise the temperature of B and lower that of A, in contradiction to the second law of thermodynamics (Maxwell, 1871, reprinted 1902).

Besides the case of enzyme recognition, the need for discrimination requiring a MxD may be illustrated in many situations. For example, it was long assumed, based on biochemical experiments, that, despite their recognized compartments (nucleus, organelles, etc.), most of what was relevant in the cell metabolism was more or less free to diffuse and spread throughout the cytoplasm without alteration. It was however critical to consider the entropy contribution of components of the cell, which required understanding their exploratory tendency in terms of time‐dependent positions and energy states. Common sense appreciates that, when significant entities are available in small numbers – a routine situation in cells – their relative position and energy states become particularly relevant. When two compounds are spatially next to one another, they can interact, and make functional complexes or non‐functional aggregates, for example. When distant, nothing happens. A relevant function is therefore to manage accurately the positioning of entities of interest. This includes taking symmetry into account, keeping energy states between narrow borders, or separating between intact or altered entities (aged ones must be distinguished from young ones in particular). In short, many critical processes are involving information management. Accurate timing also belongs to the category of events that may fit with the action of MxDs. This is the types of situation we try to clarify in the few examples detailed below.

## An open list of basic cellular Maxwell's demons

The functions we retained here are meant not only to recognize a target or an event, but, in a noisy background, to discriminate against similar targets, target states and location, or events, so that they can interact in an orderly fashion. Relevant biological interactions have, if we pursue the key/lock metaphor, to be immune against omnipresent picklocks and skeleton keys, and spontaneous, but irrelevant interactions (see the negative role of protein aggregation, for example). Natural selection operating on this need for discrimination in addition to recognition has led to functional convergent evolution of a variety of dynamic structures that behave as MxDs. The original MxD was an agent that measured properties of similar entities (gas atoms moving at different speeds) between two chambers separated by a trap opened or closed (gated) at will by the demon. Transporting molecules across membranes, when the cell must discriminate between related molecules and change their internal concentration, illustrates the embodiment of a process that is very similar to the original MxD thought experiment. We begin here with transporters that, while getting rid of waste or take in highly diluted nutrients, must take great care not to empty the cell of its important metabolites.

### ABC transporters: why hydrolysis of two ATP molecules?

Cells must import nutrients and export waste, toxic compounds or mediators of cell interactions. They must also be able to distinguish what belongs to the cell from what does not belong to it. Transport across membranes is performed via a variety of permeases [for the origin of the concept see (Buttin *et al*., 1956)]. Some are mere selective channels that allow facilitated diffusion of substrates. Using the key/lock metaphor, the substrate is the key and the permease the lock. A ubiquitous type of such permeases is the aquaporins, some of which also transporting glycerol (Ishibashi *et al*., 2017). Many other permeases ensure facilitated diffusion of their substrate (Yan, 2015). They do not use energy directly in the process. In general, however, because the cell must import compounds that are diluted in the environment, there is a free energy cost to climb up a gradient of concentration (high inside, low outside). This is critical for import of negatively charged molecules because the internal versus outside electric potential of the cell is negative. Energy is used in a variety of ways, often using vectorial transport of protons (sometimes sodium or calcium ions) into the cell (Grytsyk *et al*., 2017), or a variety of processes involving energy‐rich phosphate bonds of a coupled enzymatic reaction (Seeger, 2018).

**Figure 3:**
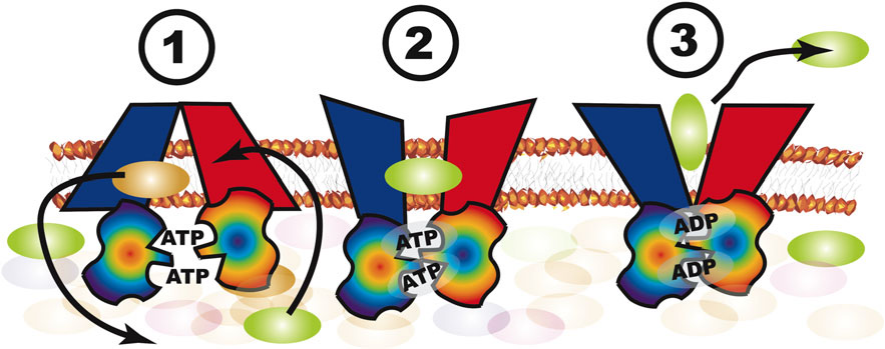
A generic ABC exporter. The relaxed state of the transporter is opened to ATP at two sites and it explores substrates to be exported (stage 1). Once a correct substrate is recognized, the transporter closes up into a tense configuration, with lowered activation energy for ATP hydrolysis (stage 2). Upon hydrolysis of ATP, the transporter opens up to the outside and releases its substrate (stage 3). Finally, the system is reset to its open configuration (stage 1). Hydrolysis of one ATP molecule is required to counteract a concentration gradient or an electric charge potential. The second ATP hydrolysis is used for information management required for discrimination of the relevant substrate against competitors.

The various types of transporters are summarized in the Transporter Classification Database (Saier *et al*., 2016). Among transporters, the so‐called ABC transporter family is dominating. The trend in publications involving these proteins kept increasing linearly since 1991. As of January 2019, some 7700 articles at PubMed were indexed using the keywords ‘ABC transporters’ OR ‘ABC transporter’. It is therefore out of the question, here, to summarize such a huge literature, which explores the mechanics of transport while emphasizing its diversity (Locher, 2016). Indeed, despite decades of work by many laboratories, the general mechanism for the transport cycle of ABC transporters is still a matter of debate. We contend that this may be due, at least in part, to the fact that the role of information management in these structures has been overlooked. To be sure, a remarkable feature of these transporters is that they bind, and then hydrolyse two ATP molecules, not just one, on two nucleotide‐binding domains [NBDs, (Wilkens, 2015)]. We try here to summarize in an algorithmic way the function cycle of these molecules, whether importers or exporters, pointing out the critical role of energy dissipation for memory erasure (Fig. 3).

The resting state of all ABC transporters has their NBDs in an open dimer configuration, usually with low affinity for ATP. Subsequently, upon binding of the relevant substrate, two molecules of ATP bind, cooperatively, to form a closed dimer configuration. The details of this sequence are not entirely clear. It seems to vary with each transporter. As discussed above, it is critical to distinguish between recognition and discrimination of the proper substrates. Curiously enough, the details of transported substrate binding have seldom been the subject of biochemical and structural analyses. A reason for this fact may be that some exporters can transport a large variety of compounds (often xenobiotics or antibiotics), apparently widely differing in their chemical properties. Yet, despite this variety, these transporters must nevertheless efficiently discriminate against the prevailing metabolites of the cell, as, otherwise, they would keep leaking out essential components. A way to do so is to tag the exported substrates with specific chemical groups, an energy‐dissipating step, allowing these transporters to act as regulatory safety valves (Danchin, 2012). Thus, we can assume that proper identification (validation) of the right substrates by the transporter is critical. As this process must be linked to an energy‐requiring informational step, we assume that it is here carried out by an ATP‐dependent process, explaining why transport requires one extra ATP instead of a single one. Indeed, preceding the binding of a substrate, ATP binding seems to be fairly weak. Upon binding of the correct substrate, a conformational change activates the dimer, which becomes ready for transport.

At this stage, the protein's conformational changes will lower the ATP hydrolysis activation barrier, preparing for the hydrolysis step that will occur later on. Subsequently (possibly simultaneously in some cases), ATP hydrolysis is coupled to mechanical changes, exposing hydrophobic residues to water (or hydrophilic residues in the lipid bilayer, for membrane lipid transporters) and opening up the transporter into the substrate release compartment (Josts *et al*., 2018). As a consequence, the substrate goes to its relevant compartment, while the transporter's conformation is relaxed, and then reset to its original open state. This reset will often be entropy‐driven because residues exposed to water will return to the inside of the protein. In parallel, phosphate and then ADP are released. It is noteworthy that this step is also a means for the cell to couple information accumulation with energy availability, measured as ADP versus ATP availability. Remarkably, it is indeed the presence of the legitimate substrate, not analogues, that triggers an ATPase activity increase, sometimes several fold over the background, demonstrating that ATP hydrolysis is coupled to selective recognition of the proper substrate. This behaviour involves a long‐distance allosteric communication that transmits the information that the substrate has bound to its relevant site, over a large distance to the site of ATP hydrolysis [for a recent example, see (Karasik *et al*., 2018)].

The need for ATP hydrolysis as an essential step for active transport has been recognized early on. It was noticed that most transporters had a background ATPase activity that was directly linked to the presence of relevant substrates [see discussion in (Beljanski *et al*., 2005)]. Unfortunately, despite the wealth of structural data – including the knowledge of substrate‐bound 3D structures – the molecular timing of substrate‐stimulated ATPase activity remains fairly elusive. In particular, the exact sequence of the hydrolysis steps for each ATP molecule is not clear as it may vary in different transporters (Seeger, 2018). Also – and this is in line with the view proposed in the present article – both ATP molecules do not seem to have the same role, despite the fact that they need both to be hydrolysed to complete a transport cycle (Kern *et al*., 2004). Some transporters have NBDs that do not have similar abilities in binding and hydrolysing ATP. Furthermore, the fact that the interface of the NBD dimer consists of two ATP‐binding pockets denotes different functions for the two NBDs in the transport cycle (Priess *et al*., 2018). Recent structural and biochemical data suggest that it is ATP binding and formation of a compact dimer in a ‘tense’ form, rather than ATP hydrolysis, that provides the ‘power stroke’ that will result in transport. This observation fits remarkably well with the fact that the informational part of the transport (discriminant recognition) should be reversible and performed without energy consumption, exactly as established in Landauer's principle. Subsequently, again in line with Landauer–Bennett's view, the system must be reset to its ground state to allow it to perform another cycle of transport. Hydrolysis of ATP follows transport and then sequential release of P_i_ and then ADP restores the transporter to its basal configuration.

In summary, one of the ATP molecules is used as the (electro)mechanical energy source required by transport (climbing up a concentration gradient or acting against a contrary electric potential), while the second one is involved in the physical management of the material system that processes information. The role of hydrolysis of two ATP molecules has been discussed in a variety of models, in particular, the alternating site hydrolysis model, proposed by Alan Senior in 1995 (Senior *et al*., 1995). Yet, the functional significance of this mechanism has eluded straightforward explanations. Here, we propose that a critical role is informational: discriminative recognition of the proper substrates of the transporter requires setting up a structure that memorized the structure of the substrate, and that, once transport has been achieved, this memory must be erased, with the transporter ready for another round of transport. This behaviour is most reminiscent of that of a Maxwell's demon that can sort gas atoms between two chambers separated by a trapdoor.

**Figure 4:**
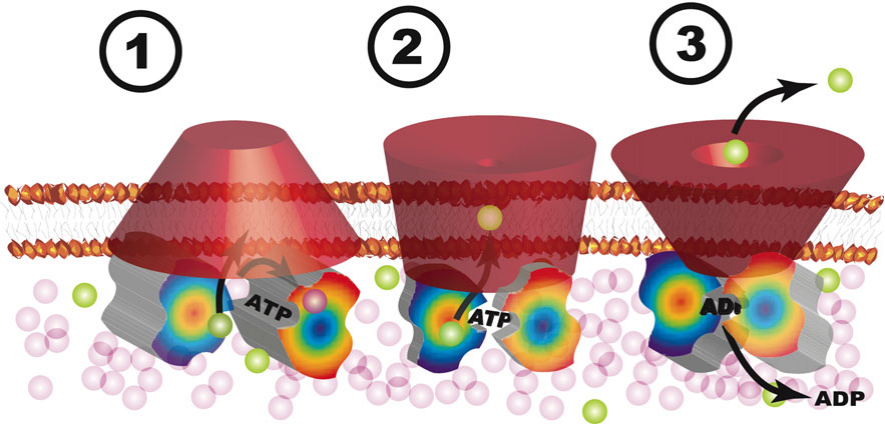
An ABC transporter degenerating into a gated pore. A pore, open to the cytoplasm, binds an ATP molecule, while allowing free diffusion of possible substrates (stage A). When the discriminated substrate encounters its binding site, the gate closes on it as well as on the nucleotide (stage 2). ATP hydrolysis relaxes the pore, which opens up to the outside, releasing its substrate (stage 3). ADP release will reset the gate to its original state (stage 1). Excess ADP will prevent release of the substrate, providing a gating mechanism tied to energy availability. If the concentration gradient or charge of the substrate favours export, then the only contribution of energy dissipation is for setting up the information‐triggered physical embodiment of Maxwell's demon.

An important prediction of the model is that, if there were conditions when the transported compound does not have to fight an electrochemical gradient (e.g. export of a negatively charged ion out of the cell), the ‘power stroke’ mechanical site would become useless and would degenerate over time. This is substantiated by experimental observations. Indeed, a variety of ABC transporters have their second ATP‐binding site degenerate. They have a role restricted to management of a gating process, which can be seen as the consequence of a ‘broken pump’ where ATP hydrolysis is now purely informational, controlling energy‐dependent gating [for example for ion transport (Linsdell, 2018)]. In the context of this work, this is an exceptionally fit illustration of the trapdoor process of a MxD, which can thus monitor the energy availability in the cell (ATP/ADP ratio, Fig. 4).

### ATP‐dependent DNA‐binding transcription regulators

A similar situation may hold for a completely different set of functions, regulation of gene expression. Indeed, while the key/lock metaphor of enzymes is easy to grasp, with diffusion of substrates as the exploratory process that guides the substrates to the enzyme active centre, the exact same view underlies the standard model of transcription control by repressor–operator interactions.

As a case in point, the LacI transcription regulator is a reversible repressor, binding the inducer allo‐lactose or related compounds. It matches exactly the key/lock metaphor, with its action following faithfully the variations in concentration of the inducer, monitored by an allosteric behaviour (Monod *et al*., 1965). However, if enzymes or regulators behaved in this way, nothing would preclude analogues – not only smaller substrates, but also all kinds of mimics – to get in and meddle with the regulation process. This is why Jacob and Monod were lucky in their choice: they could construct analogues of allo‐lactose that would fit the bill, for example the now widely used lactose operon inducer IPTG, or fail to bind, such as phenyl‐beta‐d‐galactoside (Cohen and Monod, 1957). This behaviour follows reversibly the contextual information given by the environment to the cell. No energy is involved besides that of thermal agitation. The lactose repressor works well because, in natural environments, *Escherichia coli* cells do not meet compounds that are stereochemically close to allo‐lactose. Meeting such compounds would be deleterious if transported or hydrolysed by the lactose operon products. Indeed, if this situation prevailed, then induction of the operon – which triggers a huge production of beta‐galactosidase – would be wasting important resources. This negative situation is illustrated for example in *Rhizobium meliloti* transformed with an heterologous *E. coli* lactose operon, where lactose is toxic [(Timblin and Kahn, 1984), see also (Stoebel *et al*., 2008)]. How can this hurdle be solved? There are situations when the cell will need an active discrimination process between similar compounds because there are many analogues of the proper substrate that could interfere with the process of interest, catalysis or regulation. In particular, this requirement would entail that the relevant active site of the protein can memorize the fact that it has seen the ‘right’ substrate and not analogues or mimics. It can be surmised that this will require some energy consumption. Naturally, as discussed in the introduction, erasing the corresponding memory to recycle the regulator will dissipate a significant level of energy.

With this view in mind, we can revisit the control of the maltose regulon in *E. coli*. It has long been known that, in contrast with the LacI repressor, the activator of the maltose regulon, MalT, required an ATP molecule to be properly active. This remained a puzzle for a long time. Among its fairly challenging features, MalT recognized a substrate that was initially unsuspected, maltotriose (made of three glucose subunits), in a highly specific manner (Raibaud and Richet, 1987). The underlying reason for this molecule to be involved in the control of a complex important regulon is likely because it is a marker of the ecological niche of *E. coli*. To be sure, maltotriose is abundant in mixtures that result from incomplete fermentation of plant seed mixtures [this accounts for *Saccharomyces cerevisiae* being unable to ferment maltotriose efficiently (Alves *et al*., 2018)]. Maltotriose is thus expected to be present as a major catabolic by‐product of carbohydrate fibres in the gut of many mammals, where *E. coli* thrives (van Zanten *et al*., 2012). Finally, maltotriose is also, along with maltotetraose, the main maltodextrin endogenously produced by the degradation of glycogen by the dedicated GlgX and GlgP enzymes (Dauvillee *et al*., 2005). In response to maltotriose, MalT regulates the expression of genes that are important for intestinal colonization by *E. coli* (Jones *et al*., 2008), through their role in maltodextrin catabolism and glycogen pool management (Park *et al*., 2011). In these systems, wrong decisions are presumably highly detrimental to the cell or to the multicellular organism where it multiplies. The consequence is that proper discrimination is critical. In particular, binding of substrates sharing chemical substructures with maltotriose, such as maltose or glucose, would jeopardize regulation.

How does MalT proceed? As a member of the signal transduction ATPases with numerous domains (STAND) superfamily of ATPases (Leipe *et al*., 2004), its activation mechanism relies on an ATPase core module called the NOD (nucleotide‐binding oligomerization domain). In the monomeric resting state of the protein, the NOD module adopts a closed conformation with a tightly bound ADP (Danot *et al*., 2009). Binding of the inducer (maltotriose in the case of MalT) to domains adjacent to the NOD module triggers its opening. This conformational change is required to replace ADP with ATP and results in a ‘tense’ state of the protein, competent for association into the active MalT multimer. The inducer is recognized in two steps, of which only the latter is coupled with NOD opening. The first, lower affinity‐binding step can thus be viewed as a proofreading reaction preventing improper signalling [(Danot, 2015), and see Fig. 1].

In other words, MalT can probably bind smaller substrates such as maltose or glucose, but the binding will not be tight, nor will it allow gene expression activation. Exactly as in the case of ABC transporters, activation of MalT depends on the presence of ATP together with that of maltotriose, shifting the activator to the tense state. Perhaps surprisingly at the time, when ATP was replaced by a non‐hydrolysable analogue of ATP, transcription activation was unaffected, demonstrating that ATP hydrolysis is not required for transcription activation *per se* (Richet and Raibaud, 1989). Again, this is a remarkable substantiation of the Landauer–Bennett's principle that established that creation of information is reversible. However, as expected, energy was required in the form of ATP hydrolysis into ADP and phosphate to recycle MalT back to the relaxed form, which has ADP, not ATP bound. In line with this view, a protein in which ATP hydrolysis, but not ATP binding, was abolished, could not be recycled and remained frozen in the active form (Marquenet and Richet, 2007). Whereas in ABC transporters, the activation barrier of ATP hydrolysis is lowered upon formation of the tight binding complex, here, this effect is obtained through the multimerization step (Marquenet and Richet, 2010). Whether ATP hydrolysis occurs before or after maltotriose dissociation and whether it is affected by RNA polymerase binding and transcription initiation is still a matter of speculation. Thinking in terms of MxDs should help in deciphering this sequence of events.

Bacterial STAND ATPases are regulatory proteins, either transcription factors or serine‐threonine kinases, which need to discriminate proper targets against a fairly significant noise of analogues. To date, mechanistical studies on these proteins are scarce, but typical features of the MalT activation mechanism have nevertheless been observed. In GutR from *Bacillus subtilis*, an inducer‐dependent conformational change similar to that observed in MalT was identified (Poon *et al*., 2001), while inhibition of the NOD switch by the inducer‐binding domain was suggested in AcoK from *Klebsiella pneumoniae* (Hsu *et al*., 2008). On the other hand, the ADP/ATP switch and its resetting by ATP hydrolysis and the multistep mode of recognition of the inducer followed by an ATP‐dependent multimerization appear to be conserved in a large branch of the superfamily, as judged from studies of eukaryotic members of the STAND family‐like APAF1 or the NLRC4‐NAIP immune receptors, which are also extremely discriminating towards their substrate (Yang *et al*., 2018). This suggests that other STAND bacterial activators such as AfsR from *Streptomyces coelicolor* (Tanaka *et al*., 2007), and possibly STAND serine‐threonine kinase‐like PknK from *Mycobacterium tuberculosis* (Malhotra *et al*., 2012), could also function as MxDs. Following this paradigm might also help discovery of the elusive discriminatory signals recognized by LAL regulators (large ATP‐binding regulators of the LuxR family), a poorly studied family of transcriptional regulators (De Schrijver and De Mot, 1999) that control the phosphate starvation response and antibiotic synthesis (Guerra *et al*., 2012). In the same vein, regulation of transposition of the Tn7 transposon depends on the control of TnsC, an ATP‐dependent protein that allows the transposon to discriminate between different target sites (Stellwagen and Craig, 1997). To be sure, transposon Tn7 is endowed of target immunity, a process that prevents the transposon to insert into target DNAs that already contain a copy of the transposon (Stellwagen and Craig, 2001), again a typical MxD behaviour.

### Networking: Sigma54‐dependent promoters and cognate transcription factors

Global transcription is another process that needs to be carefully controlled. Sigma factors are a family of subunits of RNA polymerase (RNAP) involved in promoter recognition. In general, there may not be strong constraints to get controls acting beyond recognition of their targets. However, under certain circumstances, turning on transcription of a large fraction of the bacterial genome would require massive deployment of resources for the formation of new gene products. It may therefore be important not only to recognize the cognate promoters, but to discriminate against noise in order to activate them only under relevant conditions, in particular while monitoring energy availability. To be sure, management of gene expression under specific environmental conditions sometimes requires discriminative power to allow the cell to express a large number of genes. One of the most remarkable cases to this end involves prokaryotic promoters dependent on the alternative and somehow unique sigma factor σ^54^ (RpoN).

Unlike the typical AT‐rich −10/−35 motifs that abound in the majority of the standard σ^70^‐dependent promoters for RNAP binding, the σ^54^‐dependent variants are notable in several respects. First, in the RNAP‐σ^54^ binding site, most conserved bases are located at positions −12 and −24 with respect to the transcription initiation site, and the region is GC‐rich. It has been generally believed that DNA strand separation required for transcription initiation from these promoters is therefore more energy demanding and thus that an extra energy must be entered in the system to trigger formation of an open complex. Furthermore, in a subset of promoters, the RNAP‐σ^54^ subunit sits on the cognate sequence forming a transcriptionally dead closed complex. While binding of typical RNAP‐σ^70^ to the −10 and −35 motifs melts DNA at the promoter to initiate transcription, typical RNAP‐σ^54^ binds −12 (GC) and −24 (GG) motifs while preventing transcription initiation at the promoter, in the absence of the matching activator. Second, the cognate transcription factors (TFs) attach to locations somewhat far from the RNAP‐σ^54^ binding site(s). This entails that productive contacts between them and the downstream‐bound polymerase require the upstream transcription factor (TF)‐DNA complex to sharply bend the intervening DNA segment. The general similarity of this scenario with that involving eukaryotic promoters run by RNAP II has popularized naming the attachment sites of bacterial TFs in RNAP‐σ^54^ complexes as ‘prokaryotic enhancers’. The corresponding proteins have been named prokaryotic enhancer‐binding proteins (EBPs) accordingly.

Contacts between the upstream‐bound EBPs and the downstream‐bound RNAP‐σ^54^ are often facilitated by the DNA‐binding protein integration host factor (IHF), which in turn imposes a fixed geometry that avoids the accidental activation of the RNAP‐σ^54^ by non‐cognate EBPs – that can also activate the RNAP‐σ^54^ holoenzyme directly, in the absence of any significant upstream DNA binding (Perez‐Martin and De Lorenzo, 1995). That transcription initiation needs a specific geometry enables the regulatory role of a large number of additional DNA‐binding proteins that either favour an optimal 3D arrangement of the initiation complex or spatially offset the key players and inhibit the process. The third remarkable feature of this family of transcriptional devices is the very organization and functioning of the EBPs. All of them include a central AAA+ domain comprising ATP hydrolysis motifs found in a superfamily of functionally highly diverse proteins (Snider *et al*., 2008). In EBPs, this module is attached to a helix‐turn‐helix (HTH) motif that secures DNA‐binding specificity. Finally, most EBPs have a hypervariable additional signal‐reception domain that ultimately triggers the whole transcription initiation process (Zhang *et al*., 2016).

A naïve, early view of the mechanism of activation of RNAP‐σ^54^ promoters argued that upon signal reception, EBPs undergo a major conformational shift that made them form stable oligomers (hexamers) that become competent for ATP hydrolysis. This process was seen as coupled to interactions with the polymerase (and otherwise dead RNAP‐σ^54^ complexes) through a conserved sequence in the AAA+ activator, known as the GAFTGA motif. This site appeared to couple ATP hydrolysis to formation of an open complex through the conserved regulatory region I of σ^54^ formation (Bush and Dixon, 2012). It would thus appear that the energy of ATP hydrolysis is just used for mechanical opening the GC‐rich sequence of the RNAP‐σ^54^ promoters. Yet, things seem to be a bit less trivial than this scenario. First, ATP hydrolysis is not necessarily required for oligomerization of EBPs, as the non‐hydrolysable variant γ‐S‐ATP triggers the process, similarly to the standard triphosphate (Perez‐Martin and de Lorenzo, 1996). Under these conditions, the succession of events is however stuck there and a further round of the oligomerization process cannot be re‐initiated unless ATP is indeed hydrolysed. A further revealing feature was unveiled by using the ATP hydrolysis transition state analogue ADP‐aluminium fluoride in an *in vitro* system with purified EBPs and pre‐opened transcription complexes (Chaney *et al*., 2001). Interpretation of the experimental results suggested that the key role of ATP hydrolysis was to allow cycles of binding and rebinding of activator and σ^54^ and not so much direct formation of a transcription open complex. This is exactly what would be expected for a MxD controlling transcription: energy‐independent discrimination of relevant targets, followed by energy dissipation to reset the system to its original state.

With this view in mind, a further aspect of the activity of some EBPs deserves a comment. A subclass of these factors binds specifically a small molecule to its signal‐reception domain (typically at its N‐terminus). Upon binding, the intramolecular repression caused by interaction of the protein module with the RNAP‐σ^54^‐activating surfaces of the AAA+ component is interrupted. Factor XylR, a TF that drives transcription of an operon for degradation of toluene in the soil bacterium *Pseudomonas putida,* is a case in point (Perez‐Martin and de Lorenzo, 1996). To initiate its control action, the N‐terminal domain of XylR must bind one of the relevant pathway's initial substrates (m‐xylene or 3‐methyl‐benzylalcohol). However, it happens that the authentic substrates of the toluene degradation operon are chemically very similar to non‐substrates, creating a need for substrate discrimination. Interestingly, a non‐substrate such as dinitrotoluene binds XylR with an affinity similar to that of a real substrate (e.g. 3‐methyl‐benzylalcohol). Yet, it seems that only a good one can activate transcription (de Las Heras and de Lorenzo, 2011), presumably by enabling the conformational transitions that allow ATP hydrolysis. It is plausible that – as was the case with the non‐hydrolysable variants of ATP in RNAP‐σ^54^ promoters mentioned above, non‐productive effectors keep the protein in a shape unable to reset its multimerization while the bona fide counterparts allow reinitiation of the cycle. This discriminative feature is typical of the action of a MxD.

Why this complexity? σ^54^ promoters seem to have been selected to control gene expression of highly regulated processes that require no expression in the off‐state, and very high expression in the on‐state. The control process entails that reinitiation of open promoter complex formation is coupled to the ATPase activity of AAA+ activator EBPs. As mentioned above, many of the features found in σ^54^‐dependent systems might be conserved in all three domains of life, specifically in eukaryotic promoters dependent on RNAP II. In terms of evolution, ancestors of σ^54^ domains may have been part of an early transcription repression system meant to keep the RNAP in check, even acting globally and in trans to genes before the RpoN domain was co‐opted for promoter‐specific binding and specific gene expression (Zhang *et al*., 2016). In these, TFs bind DNA sequences that are also distant from the promoter and DNA looping allows the activator to contact the sigma factor. This view is consistent with the fact that some σ^54^‐dependent systems also participate in inter‐kingdom signalling systems. This is illustrated in *E*. *coli* by QseF a two (now three)‐component system protein that binds upstream regions of σ^54^‐dependent promoters for synthesis of a regulatory small RNA and a second sigma factor, σ^24^ (RpoE). Associated with QseF, sensor protein QseE binds epinephrine, a metabolite that allows the system to couple the bacterial metabolism to host signals, in particular in pathogens. In this system, energy involvement is more complex than with standard EPBs, in that instead of being an ATP‐binding protein, QseF needs to be phosphorylated by QseE in complex with outer membrane sensor QseG to activate its cognate promoters. However, we witness also an energy‐dependent recycling process in that QseE must be dephosphorylated by a second activity of the QseE protein (phosphatase) to start another round of control (Gopel and Gorke, 2018).

Discriminating proper signals is particularly important for virulence, and it is probably not a surprise that there are as many as 13 bEPBs in *Salmonella enterica* serovar Typhimurium (Bono *et al*., 2017). This observation allows us to indulge in making a specific prediction, based on our view of the behaviour of MxDs. Remarkably, gene *rpoN* is encoded in Enterobacteria in an operon revealing still another level of control of gene expression that involving small regulatory RNAs (Riordan and Mitra, 2017). These RNAs interfere with a variety of essential processes in the cell where they create self‐consistent networks of regulation. Widely conserved protein RapZ is an ATP‐binding protein that channels specific regulatory RNAs (in particular GlmZ) involved in the control of aminosugar degradation to the degradosome. It has to distinguish GlmZ from other small regulatory RNAs. We speculate that this function is triggered by ATP‐dependent dimerization of the protein upon binding with its specific target – shown to result from dimerization of the C‐terminal domain of the protein (Gonzalez *et al*., 2017) – and that recycling of the protein for a new round of activity entails ATP hydrolysis, as in the other MxDs discussed previously here. This prediction could be experimentally tested.

### Accurate template recognition during translation and antibiotic resistance

Very early on, the idea that discrimination versus plain recognition was essential in biology was discussed in some molecular details. Despite the fact that, at the time, information was not yet perceived as an authentic currency of physics, Landauer's work being largely overlooked [to understand some reasons of this oversight, see (Landauer, 1975)], this notion was critical to establish the kinetic proofreading process proposed by John Hopfield and Jacques Ninio. Disregarding information but focusing on the kinetics of biochemical reactions, Hopfield remarked that we should be cautious each time we observed biological processes dissipating energy apparently in an inessential way. We should take great care in understanding the raison d’être of ‘known reactions which otherwise appear to be useless or deleterious complications’ (Hopfield, 1974). He observed that, during the mRNA translation process by the ribosome, the codon/anticodon recognition step involved energy dissipation. Yet, it might have been expected that a mere recognition by complementarity should have been sufficient. In this process, amino acid‐loaded tRNA molecules associated with GTP‐bound EF‐Tu flowed into the ribosome A site. In most cases [there are from 23 to more than 40 different tRNAs in a cell (Grosjean and Westhof, 2016)], this does not produce an accurate codon/anticodon match and the complex diffuses out of the ribosome untouched. By contrast, when the match is positive, local accommodation of the complex triggers a modification of the ribosome structure, the tRNA stays in interaction with the cognate codon of the messenger RNA while EF‐Tu hydrolyses GTP and detaches as a complex with GDP (Johansen *et al*., 2018). Subsequently, a second factor, ET‐Ts, binds to EF‐Tu‐GDP, which releases GDP, and EF‐Tu is reset to its open active state. It can then start a new cycle of aminoacyl‐tRNA binding. A somewhat similar function, discriminating initiator tRNA, involves initiation factor IF2 (Caban *et al*., 2018). The process does not appear to require a release factor in bacteria, however, but may do so in Archaea and Eukarya (Arkhipova *et al*., 2015).

At the time, this behaviour was essentially accounted for as a kinetic control system, not as specifically involved in information management. Yet, recognizing Landauer's principle, we now can see that it is exemplary of the role of a MxD, with the initial informational step suspending energy dissipation in a tense state until it is reset to its ground‐relaxed state after information has been used. The functioning of EF‐Tu is however different from that of the individual MxDs that we have met until now, as it requires the action of an accessory factor to be reset. This step introduces a remarkable versatility in the control of the availability of MxDs specific for critical processes, allowing them to be directed to specific compartments or to follow a specific timing clock rather than spontaneously organize in irrelevant interactions. We note that this family of information management by NTP‐binding proteins coupled to factors releasing NDP after the protein has carried out its function is extremely widespread, in particular in Eukarya. This is particularly prominent in multicellular organisms where information travelling through specific channels needs to be carefully compartmentalized. G‐proteins, for example, have often a very slow intrinsic GDP/GTP exchange rate, which allows fine tuning of their action by compartmentalized reset factors [see for example (Pylypenko *et al*., 2018) and references therein quoted].

Analysis of a cognate process, translation termination, suggests that specific discrimination steps may also be involved there: identification of a proper termination codon requires managing discriminating information. This, in turn, will require energy, not to get to the relevant information, but to have the machinery reset so that it can repeat its action elsewhere again. The details of the process are curiously fairly sparse, in particular in bacterial clades such as Bacilli (Tate *et al*., 2018). It may be coupled to ribosome recycling, which is indeed controlled by an ATP‐binding cassette protein in Eukarya (Imai *et al*., 2018), while it is much less well understood in bacteria (Prabhakar *et al*., 2017). However, in gammaproteobacteria or in Listeria species (but not in Bacilli), release factor 3 (coded by gene *prfC*) is an informational G protein (Baggett *et al*., 2017). Again, the process follows Landauer's principle: release factor 3 promotes recycling of release factors 1 and 2. To this aim, it binds in the GTP‐bound state and can rapidly dissociate without GTP hydrolysis from termination complex carrying release factor 1, while in the absence of release factor 1, release factor 3 is stalled on ribosomes if GTP hydrolysis is blocked (Adio *et al*., 2018).

Other translation factors may be involved as well. For example, GTPase LepA (EF4) was initially supposed to facilitate back‐translocation on the 70S ribosome, allowing the system to correct a possibly deleterious error (Evans *et al*., 2008; Liu *et al*., 2010). This function was not substantiated however and the actual function of EF4 is still enigmatic, possibly because its role in management of information has, until now, escaped attention. Although EF4 improves translational accuracy, it cannot reverse paromomycin‐induced errors. In fact, it modulates the initiation cycle of protein synthesis. It is also involved in ribosome 30S subunit biosynthesis (Liu and Chen, 2018). The informational role of EF4 is witnessed by the fact that mRNA molecules possessing strong ribosome‐binding sites tend to be translated with reduced efficiency in a *ΔlepA* background (Balakrishnan *et al*., 2014). In the same way, protein HflX is a heat‐shock‐induced GTPase that is conserved in the three domains of life (but absent from organisms with minimal genomes, see Table 1). It is a multidomain protein that possesses, in addition to its conserved GTPase domain, an ATP‐binding N‐terminal domain. This protein binds to ribosomes at the E‐site in situations of stress. It acts in splitting the 70S ribosome into its 30S and 50S components (Coatham *et al*., 2016). Binding to a sensitive form of the ribosome is associated with strong GTP binding but not GTP hydrolysis. Again this suggests, as in the previously discussed examples, that the stress‐sensing protein is in a ‘tense’ form but binds GTP in a reversible manner (Fischer *et al*., 2012), as requested for the first step of a Landauer's principle‐based MxD action. The protein also features an ATP‐binding domain that acts as a RNA helicase, unfolding regions of the large ribosomal RNA that are not properly folded (Srinivasan *et al*., 2018). Overall, the protein detects ribosomes that have been altered by heat stress and helps restore them into subunits with an active conformation. The details of the protein's function are still elusive. Taking into account its informational role as suggested here should help in improving its understanding.

**Table 1:** Maxwell's demons functions in model organisms and minimal genomes

Protein	Energy	Function	MG1655	AST	Strain 168	Syn3.0
DNA wielding						
RecA	ATP	Discrimination of ssDNA against dsDNA caused by binding of RecA to undamaged regions of dsDNA	RecA	–	RecA	*MMSYN1_0358*
RecD	ATP	Helicase‐nuclease‐mediating recognition of Chi sites	RecD	BU455	RecDB	*MMSYN1_0454*
GyrA		Topoisomerase II subunit, recognizes supercoiling	GyrA	BU180	GyrA	MMSYN1_0007
ParC		Topoisomerase II subunit, recognizes supercoiling	ParC	BU180	ParC	MMSYN1_0453
GyrB	ATP	Topoisomerase II ATP‐binding subunit	GyrB	BU010	GyrB	MMSYN1_0006
ParE	ATP	Topoisomerase II ATP‐binding subunit	ParE	BU010	ParE	MMSYN1_0452
DnaA	ATP	Recognition of OriC	DnaA	BU012	DnaA	MMSYN1_0001
PriA	ATP	Primosome helicase	PriA	BU120	PriA	–
MaoP	ATP	Chromosome macrodomain specification protein	MaoP	–	–	–
FtsK	ATP	Recombinase, topoisomerase and DNA translocase i	FtsK	–	SftA SpoIIIE	–
Lhr(RhlF)	ATP	ATP‐dependent helicase	Lhr	–	–	–
HelC(YoaA)	ATP	ATP‐dependent Chi‐dependent helicase	HelC	–	DinG	–
HelG	ATP/dATP	HelC paralogue, DNA damage inducible, activity stimulated by single‐strand DNA	HelG	–	–	–
HelD(SrjB)	ATP	ATP‐dependent DNA helicase IV involved in the RecF pathway	HelD	–	–	–
Shaping ribosomes						
BipA(TypA)	GTP	Important for the correct and efficient assembly of the 50S subunit of the ribosome at low temperature	BipA	BU433	BipA	*MMSYN1_0205*
CpgA(RsgA)	GTP	RsgA releases RbfA from 30S ribosome during a late stage of ribosome biosynthesis	CpgA	–	RsgA	MMSYN1_0263
EngA(Der)	GTP	GTPase essential for ribosome 50S subunit assembly (maturation of the 50S subunit central protoberance)	Der	BU607	Der	MMSYN1_0348
EngB	GTP	GTPase involved in ribosome 50S subunit assembly (maturation of the central 50S protuberance) and cell division checkpoint GTPase	EngB	BU432	EngB(YscX)	MMSYN1_0247
EngD	ATP/GTP	Ribosome‐associated potassium‐dependent informational ATP/GTPase	EchF	BU191	EngD	MMSYN1_0872
EraA(Bex)	GTP	Maturation of 16S rRNA and assembly of the 30S ribosomal subunit GTPase	EraA	BU257	Era	MMSYN1_0403
ObgE(CgtA)	GTP	GTPase involved in ribosome assembly and chromosome partitioning	ObgE	BU389	ObgE	MMSYN1_0377
RgpH	GTP	Potassium‐dependent GTPase involved in ribosome 30S assembly (Firmicutes)	–	–	RgpH(YqeH)	*MMSYN1_0488*
DbpA(RhlC)	ATP	ATP‐dependent DEAD‐box RNA helicase, specific for 23S rRNA hairpin 92	RhlC	–	DeaD	MMSYN1_0410
SrmB(CshA)	ATP	ATP‐dependent DEAD‐box RNA helicase required for 50S ribosomal subunit biogenesis	SrmB	BU372	CshA	MMSYN1_0410
DeaD(CshB)	ATP	ATP‐dependent DEAD‐box RNA helicase 50 ribosomal subunit biosynthesis	DeaD	BU372	DeaD	MMSYN1_0410
RhlE	ATP	DEAD‐box RNA duplex helicase;	RhlE	BU372	CshB	MMSYN1_0410
Rnr	ATP	Bifunctional 3′ to 5′ hydrolytic exoribonuclease ATP‐dependent helicase for ribosome biosynthesis and maintenance	Rnr	BU565	Rnr	MMSYN1_0775
Management of translation						
EF‐Tu	GTP	Translation elongation factor Tu	TufA TufB	BU526	TufA	MMSYN1_0151
EF‐Ts		Protein chain elongation factor EF‐Ts; resets EF‐Tu	Tsf	BU232	Tsf	MMSYN1_0539
IF2	GTP	Translation initiation factor IF2	InfB	BU377	InfB	MMSYN1_0297
RF3	GTP	RF1 and RF2 recognize stop codons and terminate translation; RF3 promotes dissociation of bound RFs	PrfC	BU543	–	–
EttA	ATP	Energy‐dependent translational throttle that gates ribosome entry into the elongation cycle	EttA	–	EttA	*MMSYN1_0118*
Uup	ATP	Nucleic acid‐binding ATPase possibly involved in translation	Uup	BU364	EttA	*MMSYN1_0118*
YbiT	ATP	Energy‐sensing translation control, ABC‐F informational factor	YbiT	–	YkpA YdiF EttM	*MMSYN1_0118*
YheS	ATP	Energy‐sensing translation control, ABC‐F informational factor	YheS	–	EttM	*MMSYN1_0118*
LepA	GTP	Modulator of translation GTPase	LepA	BU260	LepA	MMSYN1_0285
HflX	GTP/ATP	Ribosome‐splitting GTPase; ATP‐dependent helicase	HflX	–	HflX	–
RNA wielding						
RhlB	ATP	DEAD‐box helicase; component of the degradosome, associates to polynucleotide phosphorylase	RhlB	BU372	CshA DeaD	–
HrpA	ATP	Processing of fimbrial mRNA operons	HrpA	–	–	–
HrpB(YadO)	ATP	ATP‐dependent helicase; modulation of pili expression	HrpB	–	–	–
RapZ	GTP(ATP)	Adaptor for sRNA degradation	RapZ	–	YvcJ	–
Shaping membranes and cell division						
Ffh	GTP	Signal recognition particle GTPase	Ffh	BU393	Ffh	MMSYN1_0360
FtsY	GTP	GTP hydrolysis required for proper localization; binds ribosomes in a SRP (Ffh+Ffs) particle and GTP‐dependent manner, releasing trigger factor (Tig) from the ribosome	FtsY	BU024	FtsY	MMSYN1_0429
FtsA	ATP	Cell division and septation protein; recruited to FtsZ ring	FtsA	BU213	FtsA	MMSYN1_0523
FtsE	ATP	Antagonizes FtsA polymerization to promote schizosome assembly; ATPase mutants of FtsEX block activity by locking FtsA in the inactive form or preventing FtsA from communicating with other schizosome proteins	FtsE	[BU296]	FtsE	MMSYN1_0707
FtsX		FtsEX membrane‐binding subunit	FtsX	–	FtsX	ambiguous identification
FtsZ	GTP	Septal ring GTPase required for cell division and growth; dynamic treadmilling (mechanical action)	FtsZ	BU212	FtsZ	*MMSYN1_0522*
SecA	ATP	Protein targeting to the SecYEG and YidC machinery; possibly mechanical action	SecA	BU201	SecA	MMSYN1_0095
Chaperones						
DnaK	ATP	Molecular chaperone Hsp70, ATPase	DnaK	BU153	DnaK	MMSYN1_0542
DnaJ		Co‐chaperone Hsp40	DnaJ	BU152	DnaJ	MMSYN1_0541
GrpE		Nucleotide exchange factor for the DnaKJ chaperone	GrpE	BU184	GrpE	MMSYN1_0543
GroEL	ATP	Hsp60 ATP‐dependent chaperonin	GroEL	BU019	GroEL	–
GroES		Co‐chaperonin binding to Hsp60	GroES	BU018	GroES	–
HtpG	ATP	Co‐chaperone Hsp90; interacts with DnaK and RpoH	HtpG	BU483	HtpG	–
ClpB(htpM)	ATP	Molecular chaperone protein, disaggregase, Hsp104	ClpB	–	ClpC ClpE	MMSYN1_0545
HscA	ATP	Chaperone with ATPase activity for iron–sulfur cluster management	HscA	BU605	–	–
HscB		Co‐chaperone for iron–sulfur cluster management	HscB	BU604	–	–
Discriminative proteases						
ClpX	ATP	Molecular chaperone, unfoldase, funnels unfolded proteins into ClpP	ClpX	BU476	ClpX	–
ClpA	ATP	Specificity factor overlaps with ClpX in recognition of the SsrA tag	ClpA	–	ClpC ClpE	MMSYN1_0545
ClpP	ATP	Protease subunit associated to ClpX	ClpP	BU475	ClpP	–
ClpY(HslU)	ATP	Specificity factor of protease ClpYQ	ClpY	BU579	ClpY	–
ClpQ(HslV)		Protease subunit associated to ClpY	ClpQ	BU578	ClpQ	–
Lon	ATP polyP	Processive protease	Lon	BU477	LonA LonB	MMSYN1_0394
FtsH	ATP	Integral membrane ATP‐dependent zinc metallopeptidase; complexed with HflCK; degrades SsrA‐tagged proteins in the periplasm	FtsH	BU382	FtsH	MMSYN1_0039
HflC		Modulator of FtsH activity	HflC	BU567	–	–
HflK		Modulator of FtsH activity	HflK	BU568	–	–

The table lists Maxwell's demons identified in *E. coli* MG1655 and *B. subtilis* strain 168. For comparison, the presence of these functions is identified in *B. aphidicola* AST and *M. mycoides* Syn3.0. When italicized, a function from the parent strain *M. mycoides* Syn1.0 has been found to be dispensable in Syn3.0. A limited set of examples of MxD that are not conserved in minimal genomes is also presented.

Further informational processes impact translation. For a long time, characterizing an enigmatic class of soluble proteins that displayed all the main features of ABC transporters, including the presence of two ATP‐binding sites, remained a challenge. This class of ABC proteins, which belong to the ABC‐F family (Davidson *et al*., 2008; Boel *et al*., 2014), has been identified as comprising authentic translation factors (Boel *et al*., 2014). Hitherto, it has been found that they all use ATP upon binding to the E‐site of the ribosome. Several ABC‐F factors have been studied some in detail. Strikingly, a single functional ATP‐binding site was necessary for the factor to bind ribosomes. Subsequent hydrolysis of one ATP molecule was necessary to release the bound factor, an action that we can now interpret as required to reset the system to its ground state. A protein of this family, EttA (energy translation throttle A), has been characterized in further details. It inhibits the first round of elongation after translation initiation of a family of mRNA molecules. Remarkably, the first step of EttA's action is reversible. When the cell's energy level is low, it keeps the mRNA in a dormant state, bound to the ribosome at the initiation stage. As expected, following Landauer's principle, this implies that EttA is poised to sense any fluctuation in the ATP concentration, possibly using its second ATP‐binding site to set in motion relevant mRNA translation. As a consequence, EttA is able to gate the entry of specific initiation complexes into the elongation cycle as a function of energy availability in the cell. EttA binds exclusively to translation initiation complexes. This implies that the protein needs to distinguish ribosomes carrying a charged initiator tRNA in the P‐site bound to its cognate mRNA, from other ribosomal initiation complexes. As a consequence, but this is not yet demonstrated, EttA could target a sub‐category of translation initiation complexes.

Several EttA‐like proteins (Uup, YbiT, YheS) exist in *E. coli*, but their function is not yet understood. There are counterparts in *B. subtilis* (YkpA, YdiF, EttM) and at least one copy in the streamlined genomes of *Buchnera aphidicol*a and *Mycoplasma mycoides* (Table 1). Similarly, in Archaea and Eukarya, ribosome recycling is coordinated by a succession of ATP‐dependent steps that are mediated by a protein, ABCE1, coupling two ATP‐binding sites, that follows a sequence of transformations highly reminiscent of the behaviour of EttA (Gerovac and Tampe, 2018; Nurenberg‐Goloub *et al*., 2018). Other cytoplasmic ribosome‐binding ABC‐F proteins (e.g. VmlR in *B. subtilis*) have been found to endow cells with antibiotic resistance (Crowe‐McAuliffe *et al*., 2018), and this opens up novel target for discovery of novel antibiotics. However, the details of the mechanism by which they mediate resistance remain unclear. They are thought to bind to antibiotic‐stalled ribosomes and promote dissociation of the relevant compound from its binding site. They use one ATP molecule to bind to their target and require hydrolysis of another ATP molecule to dissociate from the ribosome. Here again, one of the ATP molecules could be used to reset of the system after the protein has identified the ribosomes inhibited by the antibiotic. To be sure, experimental evidence has established that antibiotic resistance ABC‐F proteins reset the peptidyl transferase centre of the ribosome to counter translational arrest (Crowe‐McAuliffe *et al*., 2018; Murina *et al*., 2018).

### Other processes working out specific features of nucleic acids

Chromosomal DNA is a long protein that folds back on itself multiple times to be accommodated in the small compartment that makes the cytoplasm (or the nucleus in Eukarya). This implies that many structural features of the molecule must be supervised not only during replication (with a need for identification of the origin and termini of replication, for example), but also as transcription develops while a variety of accidents are doomed to happen. As cases in point, DNA strands may be broken and this will alter supercoiling with a considerable change in DNA compaction. Broken ends must be repaired, and this implies recombination with intact segments of available DNA molecules. Keeping up with the theme of this article, we now look for processes involving energy in an unexpected or unexplained way to identify such control systems. Important nucleic acid‐binding proteins displaying an enigmatic ATPase activity could indeed now be seen as a MxD that recognizes specific DNA features. We document here two cases, that of the recombination protein RecA, and that of topoisomerases of class II. We also provide hints about further processes that are likely to require the action of MxDs, in particular replication initiation and termination.

#### A short travel in recombination

Protein RecA, a key partner of the recombination process [*recA* null mutants are viable however, and the protein is dispensable in *M. mycoides* Syn3.0 (Hutchison *et al*., 2016) and Table 1], has long been known to be a DNA‐dependent ATPase. Yet, its initially recognized functions, homology search and strand exchange activities, seem to be uncorrelated to its ATPase activity. RecA binds to regions of damaged DNA to promote recombination and repair. To this aim, RecA protomers assemble into clusters at nucleating sites, making filaments on the single‐strand DNA regions formed at sites of double‐strand DNA breaks or stalled replication forks. This forms a presynaptic complex. Each cluster grows to cover the single‐stranded DNA segment. However, because there is multiple nucleation, this process may leave short gaps between the clusters. Closing gaps between two adjacent clusters require some ATP hydrolysis, associated with recognition of recombination conserved regions, Chi sites (GCTGGTGG in *E. coli*), RecA proteins recognizing and self‐aligning to a 3‐nt‐period sequence pattern of TGG (Kim *et al*., 2017). This requirement for ATP hydrolysis is however quite limited, implying that another critical energy‐dissipating process must be mediated by RecA. As a matter of fact, another protein complex, RecBCD, which is an ATP‐dependent helicase–nuclease complex, is the system that mediates recognition of the Chi sites (Amundsen and Smith, 2019), thus exhibiting a MxD‐like behaviour.

It now appears that strong ATP hydrolysis by RecA is actually linked to a previously overlooked function of the protein. The relationship between RecA's ATPase activity and its DNA‐binding affinity, together with the demonstration that strand exchange can occur in the absence of ATP hydrolysis, led to the proposal that a function of RecA's ATPase activity is to release the protein from the products of strand exchange, thereby making the hybrid intermediate available to late‐acting recombination proteins. With this view, the critical role of RecA's ATPase activity is to prevent accumulation of toxic dsDNA complexes caused by direct binding of RecA to undamaged regions of dsDNA (Gataulin *et al*., 2018). Here, we have an ATP hydrolysis‐dependent error‐correction function that allows cells to discard the dead‐end and potentially toxic complexes that result from direct RecA binding to dsDNA. The authors of this work suggested that this type of error‐correction process differed from the classical kinetic proofreading mechanism. Yet, in the context of the present article, allowing discrimination of ssDNA against a large excess of dsDNA can be seen as formally similar to the codon/anticodon discrimination process. When RecA is involved in resolving presynaptic processes, it goes through a step where it must remain for some time bound to dsDNA. It is therefore essential that discrimination between ssDNA and dsDNA binding (both possible) is drastically in favour of ssDNA binding. This is where the action of RecA as a MxD is required. Discrimination involves a tense form of ATP‐RecA‐substrate binding. If the substrate has to be discriminated against, then the RecA‐ATP complex is hydrolysed to RecA‐ADP and phosphate, and then, ADP is released and resetting RecA is restored in its original form.

In the same vein (Table 1), several other *E. coli* ATP‐dependent helicases that may act as MxDs for discrimination of specific targets are also involved in a variety of recombination processes. Lhr(RhlF) is a large helicase of unknown specificity (Ejaz and Shuman, 2018) also active in *M. tuberculosis*, possibly involved in site‐specific recombination (Ejaz *et al*., 2018). HelC(YoaA) directs repair factors to a blocked DNA fork (Brown *et al*., 2015). HelG is a paralogue, whose ATPase activity is stimulated by single‐stranded DNA (Cheng and Wigley, 2018). Finally, HelD is a dispensable ATP‐dependent DNA type IV helicase involved in the RecF recombination pathway (Buljubasic *et al*., 2012). This list illustrates, in *E. coli*, the fact that many MxDs have been created in the course of evolution. This general function is expected to have the number of its representative steadily increasing as we analyse the behaviour of new species.

#### ATP‐dependent (type II) topoisomerases

Type II topoisomerases change DNA topology by passage of one DNA duplex (named the transfer, T‐segment) through a transient double‐stranded break in another duplex (named the gate, G‐segment). These enzymes ensure that supercoiling of DNA fits with its requirement as a template for efficient transcription. This implies that topoisomerases measure supercoiling locally, then modify it, if not fitting. The spatial structure of a variety of these enzymes is known and used to infer their mechanism of action (Morais Cabral *et al*., 1997). We describe the situation with DNA gyrase (GyrA/GyrB), but the situation is similar with topoisomerase IV paralogues ParC/ParE [see mechanisms of resistance to quinolones, e.g. (Kawai *et al*., 2015)].

In a first step, the enzyme cuts the DNA duplex on both strands, creating a ‘gate’ between the double‐strand breaks that are maintained in place by covalent binding to the enzyme. At this stage, a first ‘quantum’ of energy, resulting from ATP hydrolysis, is used to create the gate. Subsequently, another segment of the DNA molecule (that needs to be located in proximity of the DNA‐gate) is transferred through the gate, which is subsequently sealed back into a continuous DNA duplex (Belotserkovskii *et al*., 2006). In the course of this process, an extra ATP molecule is hydrolysed, not simply a single one. Despite decades of work, the details of the process are not entirely understood. This is because standard structural techniques do not allow witnessing movements that must be inferred from a succession of static images.

**Figure 5:**
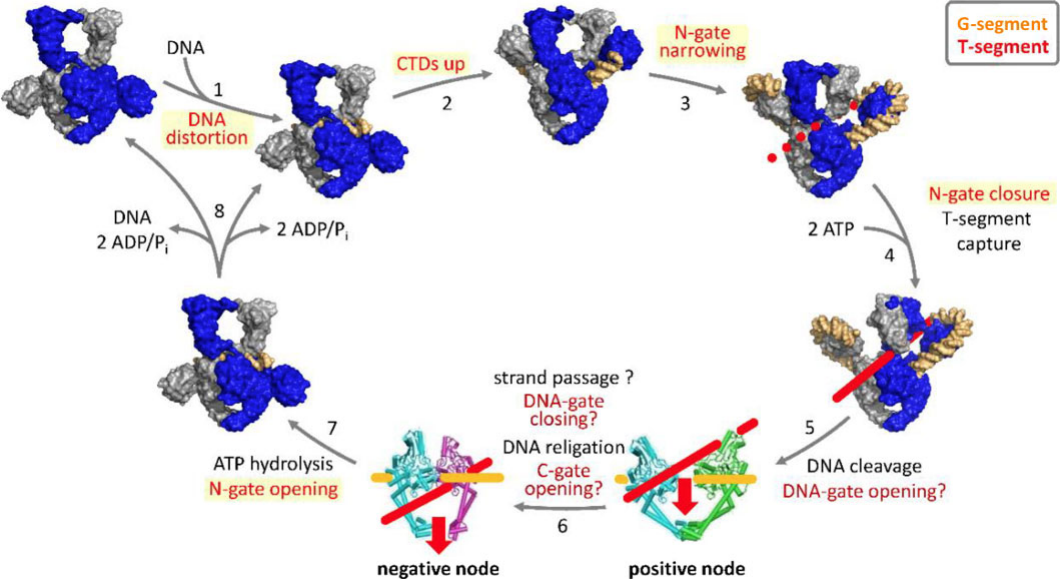
Management of negative supercoiling by DNA gyrase [figure and legend from fig. 3 in (Klostermeier, 2018), see text]. Gyrase binds the G‐segment (orange) at the DNA‐gate (1). DNA contacts with the carboxy‐terminal domains (CTDs), causing the CTDs to move upward (2). Wrapping of the DNA around the CTDs leads to narrowing of the N‐gate (3). Nucleotide‐induced N‐gate closure fixes the T‐segment (red) above the G‐segment (4). Cleavage of the G‐segment and DNA‐gate opening (5) would then enable strand passage (6), which converts the DNA bound in a positive node into a negative node. DNA‐gate closure allows the G‐segment to be re‐ligated. The T‐segment could then exit from the lower cavity though the open C‐gate (6). ATP hydrolysis leads to re‐opening of the N‐gate (7). After product release (8), gyrase is reset for subsequent catalytic cycles. Postulated conformational changes are labelled in red, and those demonstrated by single‐molecule FRET are highlighted by a yellow box. DNA‐ and C‐gate opening as well as strand passage have not been observed experimentally and are labelled with question marks. One GyrA and one GyrB subunit of the gyrase heterotetramer are depicted in blue, and the second GyrA and GyrB subunits are shown in grey. The red dashed line in the gyrase/DNA complex after N‐gate narrowing (3) indicates the extrapolated T‐segment. In the crystal structures of GyrA dimers with open DNA‐ or C‐gate (between steps 5 and 6, and 6 and 7 respectively), one GyrA subunit is shown in cyan and the second in green or magenta. The red arrows mark the passage of the T‐segment through the gap in the G‐segment and the open DNA‐gate, and through the open C‐gate respectively.

The gating process can be further split into three stages. Type IIA topoisomerases form a symmetric structure with three protein/protein interfaces, termed N‐gate, DNA‐gate and C‐gate through which a DNA segment must be transferred. The G‐segment DNA is broken in two at the DNA‐gate, creating a physical gate. At this point, the enzyme has to manage information, recognizing conditions where it is appropriate to capture a T‐segment in a particular topological organization in order to move it through the DNA‐gate. The enzyme has to evaluate DNA topology locally, where it binds. This is an information‐measuring process. As discussed previously, this process will dissipate energy: two ATP molecules are used, one to measure supercoiling and one to transport the segment through the gate, in a behaviour quite reminiscent of that of ABC transporters. When measuring a proper conformation, ATP binding results in N‐gate closure, fixing the T‐segment above the G‐segment in the upper cavity of the enzyme. Subsequently, the DNA‐gate opens up, the T‐segment goes through it, while the C‐gate opens and releases the T‐segment from the bottom cavity. Hydrolysis of both ATP molecules leads to re‐opening of the N‐gate and resets the enzyme for subsequent catalytic cycles [Fig. 5 reprinted from fig 3 of (Klostermeier, 2018)]. We should note here that the very fact that this behaviour is reminiscent of a MxD has been pointed out previously (Rybenkov, 2016). This previous analysis however overlooked reference to the obvious adequation of this system to Landauer's principle.

#### Initiation and termination of replication

Other processes involved in replication also require efficient discrimination of the target site among a large number of similar sites. This is the case for example of replication initiation, where protein DnaA, a member of the AAA+ superfamily, has to recognize accurately an origin in the chromosome. This function has long been known to be ATP‐dependent. Yet the role of ATP is poorly understood (Hansen and Atlung, 2018). The function of AAA+ superfamily proteins is, indeed, generally interpreted as mechanical (Snider *et al*., 2008). We propose that DnaA works as a MxD meant to recognize OriC sequences while discriminating them against similar sequences elsewhere in the genome. Overall, initiation of replication might require both MxDs and mechanical energy‐dependent subunits. To be sure, replication initiation involves a multiprotein complex, with, beside DnaA, helicase PriA as another ATP‐dependent factor that could act as a further discriminating MxD or possibly as a mechanical transducer (Windgassen *et al*., 2018). DNA primase, that starts replication, might even combine recognition of the target initiation site with formation of a first dinucleotide primer made from the two ATP molecules used in the process, an economical way to make use of ATP in the process (Boudet *et al*., 2019).

With the size of genomes increasing, the urge for MxDs allowing the organization of genomes into domains became evident. As a case in point, the genome of *E. coli* is organized into several macrodomains that are visible using microscopic techniques. This prompted us to look for emergence of DNA domain‐specific MxDs. It seems that protein MaoP(YifB), belonging to the AAA+ superfamily, could illustrate such a function. The MaoP/maoS system (maoS are specific 17‐bp sequences recognized by MaoP, restricting DNA mobility in adjacent regions) is responsible for constraining DNA mobility in the Ori region, limiting long‐distance DNA interactions with other regions of the chromosome. Macrodomain organization influences the segregation of sister chromatids and the mobility of chromosomal DNA (Valens *et al*., 2016). We can expect the frequent presence of similar MxDs in other genomes as well (PP_5239 is a counterpart in *Pseudomonas putida*), possibly resulting from convergent evolution. Interestingly, MaoP coevolved with Dam methylase as well as SeqA, MukBEF and MatP that are all involved in the control of chromosome conformation and segregation.

To be sure, chromosome segregation in the right compartment is yet another line where management of information is essential. Another AAA+ superfamily protein involved in cell division has previously been suggested to behave as a MxD (Donachie, 2002). Protein FtsK activates XerCD‐dependent recombination. Under the form of an hexameric ring, it also links chromosome segregation and cell division in *E. coli*. Ring formation and translocase activity require cleavage by the recombinase subunits. Yet, similar to the multiplicity of RecA functions, these functions may be accessory to the key function of the protein: forming a hole at the division interface with DNA travelling through the central hole and controlling DNA segregation from the parent to the daughter cell (Donachie, 2002). Its counterpart in *B. subtilis,* SftA, is a DNA‐translocating ATPase that, prior to septum closure, moves non‐segregated DNA into opposite cell halves, as would a MxD do (El Najjar *et al*., 2018). An homologue of SftA, SpoIIIE, is further involved in parallel in chromosome segregation between the cytoplasm and the spore in statu nascendi. Thus, the details of information management of chromosome segregation at the cell's poles remain open to speculation. It certainly requires mechanical energy, but management of information is also more than likely, suggesting that at least some of its NTP‐dependent components are MxDs.

We note that four *B. subtilis* chromosome segregation proteins possess an ATP‐binding Walker motif: the bacterial tyrosine kinase (BY‐kinase) PtkA, the chromosome segregation protein Soj (ParA), the cell division protein MinD and a transcription regulator, SalA. These proteins appear to have arisen via duplication of an ancestral ATP‐binding domain fused with other functional domains. Despite having very different physiological roles, these four proteins engage in a high number of binary functional interactions allowing them to reach proper locations. MinD attracts Soj and PtkA to the cell pole, and, in addition, activates the kinase function of PtkA. SalA also activates the kinase function of PtkA, and it gets phosphorylated by PtkA as well. The consequence of this phosphorylation is the activation of SalA as a transcriptional repressor. SalA also requires ATP binding to recognize its proper target, ScoC, which represses synthesis of extracellular proteases, and bacilysin synthesis and sporulation (Barbieri *et al*., 2016). Its effectors are poorly known at this time. Derouiche and co‐workers hypothesized that these functional interactions remained preserved during evolution, representing a constraint on the process of evolutionary ‘tinkering’, brought about by fusions of different functional domains (Derouiche *et al*., 2016). In the context of the present work, the evolutionary constraint would be that of management of information.

During *B. subtilis* sporulation, the mother cell and the forespore follow different development programmes that link signal transduction pathways to compartmentalized gene expression. Shortly before the end of the process, a signalling pathway originating from the forespore triggers the proteolytic activation of the mother‐cell transcription factor sigma K generated from a precursor. Cleavage of pro‐sigma K to its mature form is catalysed by the membrane metalloprotease SpoIVFB. This protease is present in the mother‐cell membrane that surrounds the forespore and is maintained inactive by two mother‐cell membrane proteins, SpoIVFA and BofA. Activation of pro‐sigma K processing requires a second protease, SpoIVB, secreted from the forespore into the intercompartment space. SpoIVB cleaves the extracellular domain of SpoIVFA. Despite decades of work, however, how this cleavage activates intramembrane proteolysis is still a matter of controversy. Recent work – an *in vitro* analysis and a reconstitution in *E. coli* – demonstrated that this information‐channelling process was ATP‐dependent (Zhou *et al*., 2013; Halder *et al*., 2017). Yet, a genetic analysis of the corresponding sequence of events failed to identify any role for ATP hydrolysis (Ramirez‐Guadiana *et al*., 2018). However, the authors proposed that the process follows a substrate‐gating model (obviously entailing management of some information) where SpoIVFA and BofA inhibit SpoIVB by stabilizing a closed, substrate‐inaccessible conformation that prevents pro‐sigmaK access to the catalytic centra of the protease. Subsequently, SpoIVB‐dependent cleavage of SpoIVFA destabilizes this conformation leading to lateral displacement of the first and sixth transmembrane segments of the membrane‐embedded protease. This is thought to allow access of the pro‐sigma K domain to the catalytic centre of the enzyme. How cleavage of SpoIVFA triggers the open conformation is still unknown, but in the context of the present work, we can speculate that this is at this stage that ATP is used, initially bound (but not hydrolysed) to the tense closed state and subsequently hydrolysed to reset the system in its original relaxed open conformation.

### Positioning structures correctly

Cells, bacteria included, are not a bag of ribosomes, enzymes and metabolites. All entities fall into specific compartments, often defined by a protein, a lipid structure or, as recognized recently, sometimes defined by the formation of phases constructed via microscopic interactions between relevant partners (Langdon and Gladfelter, 2018). The ubiquitous compartmentalizing structure in bacteria is the cytoplasmic membrane. There are other compartments (outer membrane, periplasm, internal membranes in cyanobacteria, carboxysomes, etc.), and proper targeting of proteins is an essential feature of the cell's life. The items that collectively allow the cell to function properly must follow an accurate blueprint defined by the genetic programme. Among other features, this results in shaping the cell into a unique species silhouette, usually bacillus‐like but often following a large variety of forms (Kysela *et al*., 2016). Another spatial constraint implicates metabolism. To prevent metabolic accidents caused by the mistaken placing of reactive compounds (Danchin, 2017), metabolism must be channelled into highly specific compartments (de Lorenzo *et al*., 2014). Briefly, the way compartments are created and managed entails creating and managing information, not only plain matter, as Athenians recognized more than two millennia ago when they kept Theseus’ ship afloat despite the weathering of its wood boards [Danchin, 1998, translated in (Danchin, 2003)].

#### Defining the cell's shape

Membrane lipids are closely associated with compartmentalization. Specific phospholipids such as cardiolipins are clustered at curved regions of the membrane, often the cell's poles, and this is taken as a reason for their proper positioning (Laloux and Jacobs‐Wagner, 2014). These regions are also associated with replication. As a case in point, DnaA colocalizes with cardiolipins (Saxena *et al*., 2013). How cardiolipins get to the poles asks a chicken and egg question. They are both necessary to curve membranes and to position cardiolipin synthase. For example, ClsA colocalizes with its cardiolipin products (Romantsov *et al*., 2018). In the same way, cardiolipins are closely associated with cristae in mitochondria (Ikon and Ryan, 2017; Schlame and Greenberg, 2017; Oemer *et al*., 2018). These molecules can act as seeds to induce local curvature in an autocatalytic way. As a case in point, cardiolipin synthase is required for *Streptomyces coelicolor* morphogenesis (Jyothikumar *et al*., 2012). However, to prevent formation of more or less randomly distributed curved regions – which certainly happens with cells having astrocytic shapes, for example – it is necessary that the positional information is memorized, preventing formation of spurious pseudo‐poles and maintaining those that are retained as functional.

Within the frame of the present work, pointing out that information maintenance will require MxD‐like nanomachines, we may investigate whether such functional entities could indeed be identified in the formation of the cell's poles for example. Among many possibilities, oscillating factors such as MreB actin‐like protein define the poles in association with a variety of other factors (Colavin *et al*., 2018; Fu *et al*., 2018; Shi *et al*., 2018). The details of shape formation would require specific factors acting as MxDs. To identify (some of) those, we may look into the large number of energy‐dependent cofactors of MreB that exist in different species. The first line of evidence that would identify this behaviour is NTP hydrolysis, usually via an ATPase or a GTPase activity.

#### Managing cell division

Poles display positional information used by a variety of organizers in different bacteria, as expected for differential information management of the cell's shape when it divides. The details of the process depend on the species. Proteins such as DivIVA in Firmicutes and Mollicutes, HupP in *Vibrio cholerae* or TipN and PopZ in *Caulobacter crescentus* are obvious candidates that get to poles, but how are poles maintained? While many proteins appear to cluster in an orderly fashion around these organizers, a common quality remains to be fully understood. Several ATPases are involved in the process. ParA is an oscillating ATPase, hence likely using ATP for mechanical energy management. The polar targeting of ParC and FlhG by HubP appears to rely on different mechanisms, and a distinct region of HubP is needed to localize each client protein goes to the pole (Takekawa *et al*., 2016), which implies that the pole has been previously defined. A unifying informational role of ATPase FtsA, which has features in common with ABC‐F proteins, should be further explored in‐depth, as variants of this protein result in gain of function with alteration of the cell's shape, a behaviour consistent with a role as a MxD. This happens in an ATP‐dependent manner, in line with management of information for shaping the cell and controlling to cell division (Schoenemann *et al*., 2018). FtsA is recruited to a ring of FtsZ proteins that use their GTPase activity for energy‐dependent treadmilling, a mechanical activity (Du *et al*., 2018). In parallel, an informational complex, FtsEX, also based on an ABC‐F‐like structure, antagonizes FtsA polymerization and finely tunes the cell's morphogenesis (Meier *et al*., 2017).

Interestingly, the MxD analogy has been used previously, not when accounting for information management, but rather when discussing the need for mechanical processes in nanomachines, in particular in formation of structures such as viral capsids or DNA packaging (Bustamante *et al*., 2011). In many biological processes, in particular those using AAA+ superfamily proteins (Wang, 2004), the overlap between requirement both of mechanical energy and information management probably accounts for the blind spot that seems to have hidden the ubiquity of Landauer's principle. We discuss further examples of this state of affairs below and finally give a quantitative evaluation of the contribution of information‐related energy compared to mechanical energy.

#### Targeting proteins to the membrane

Yet another mechanical process possibly involving MxDs is targeting protein to membranes. To this aim, bacteria use several parallel protein transport systems. The majority engage in two distinct systems, depending on their connection to the translation machinery. The first one targets proteins co‐translationally into the membrane, using two universally conserved GTPases, the signal recognition particle (SRP) and its receptor FtsY, which deliver inner membrane proteins to either the SecYEG translocon or the SecX(YidC) insertase for membrane insertion. The very fact that this system is involved in recognition of specific signals substantiates its role as an information‐management system. Indeed, the recognition step of the system is triggered before GTP hydrolysis, yet another example of energy‐independent (reversible) creation of information. Efficient targeting relies on membrane interaction of FtsY and heterodimerization with the SRP protein Ffh, in a way that fits well with Landauer's principle, with GTP hydrolysis used to reset the system to its ground state ready to accept a new polypeptide in statu nascendi for membrane insertion (Kempf *et al*., 2018).

The second system depends on the ATPase SecA that targets proteins designed to be exported – periplasmic and outer membrane proteins in diderms – to SecYEG. There, the role of ATP hydrolysis is still a matter of speculation. It has been described in structural terms (Chada *et al*., 2018), but whether it is involved in a mechanical translocation process – the most common view of its role (Maillard *et al*., 2011; Ernst *et al*., 2018) – or in recognition (or both) remains to be firmly established. Export of proteins via the SecYEG machinery depends on the membrane proton‐motive force and the exact role of ATP hydrolysis, while accepted as mechanical (Fessl *et al*., 2018), could still be informational. Were this role be mechanical, one would expect a stoichiometry of ATP hydrolysis considerably higher than one for each secreted protein. Also, this stoichiometry would be expected to depend on the size of the secreted protein. In any event, while SRP selects its substrates already very early on during their synthesis, the recognition of secretory proteins by SecA is believed to occur primarily after translation termination, i.e. post‐translationally (Steinberg *et al*., 2018).

### Shaping cytoplasmic structures

Besides the shaping of the membrane, many organelles in the cell must be shaped properly while being targeted to specific locations. The ribosome is a key nanomachine that must follow a stringent construction process. In particular, proper RNA folding, especially under conditions where G–U pairs are acceptable in addition to G–C pairs, opens up a gigantic number of folding possibilities [see (Schroeder, 2018) for an up‐to‐date view of the problem]. It is therefore expected that specific systems will channel assembly towards a limited number of folded structures, identifying, then eliminating, non‐functional alternatives. This entails discrimination of what a correct structure is, with respect to an ocean of similar incorrect ones. These processes are expected to be extremely temperature‐dependent.

#### Ribosome formation and assembly

The ribosome, a ribonucleoprotein particle that is used to ‘read’ the message carried over from a DNA segment into a messenger RNA molecule, is the most important entity of the cell. This organelle is a ribozyme endowed of peptidyl transferase activity. In bacteria, it is made of three long transcript precursors that are folded and matured into 16S RNA, 23S RNA and 5S RNA molecules. These molecules further interact with ribosomal proteins that act as scaffolds and consolidating agents, based on a lock‐and‐key type of interaction, complemented with local entropy‐driven folding of disordered or flexible regions (Keul *et al*., 2018). As in enzyme catalysis, where substrates must meet an enzyme to allow it to perform a catalysed reaction, this process is essentially driven by diffusion. This simple biogenesis scenario was long prevailing, until it appeared that many other proteins, often less dispensable than ribosomal proteins especially under non‐standard temperature conditions, were required to yield a functional ribosome. Remarkably, besides enzymes that were meant to modify nucleotides, locking‐in functional RNA structures, these unexpected proteins had in common the fact that they were GTPases (sometimes ATPases). Also, they appeared to have a truly essential function. Their role was not structural, as they were not found in mature ribosomes in a stoichiometric amount. Why did not they behave as ‘standard’ ribosomal proteins?

It is not possible within the scope of this article to provide details of the function of each of the proteins involved. Table 1 summarizes the situation as understood at this time. At least seven GTPases are necessary to allow the cell to construct functional ribosomes. Interestingly, these enzymes, that we see here as examples of MxDs, are likely RNA chaperones. They are conserved in minimal genomes such as that of *B. aphidicola* (Silva *et al*., 2001) or the synthetic form of *M. mycoides*, strain Syn 3.0 [(Hutchison *et al*., 2016), as further annotated in Ref. (Danchin and Fang, 2016)]. Thus, their function does not seem to be dispensable. A core set of related GTPases (BipA, CpgA, EngA, EngB, EngD, EraA, ObgE), which cannot substitute for one another, is required to construct a fully active ribosome (Table 1).

In addition to these essential proteins, several ATP‐dependent helicases with somewhat overlapping activities are also necessary for ribosomal RNA maturation, ribosome assembly and misshaped ribosome degradation. They belong to the DEAD‐box ATP‐dependent RNA remodelling factors. They have somewhat redundant activities (Redder *et al*., 2015). Actual functional identification of the proteins required for ribosome biogenesis is therefore equivocal. As an illustration of their behaviour, helicase SrmB(RhlA) participates in the assembly of the large ribosomal subunit in *E. coli* by facilitating the incorporation of L13, one of the ribosomal proteins that bind 23S rRNA earliest. SrmB is tethered to nascent ribosome through interactions with L4, L24 and the region from domain I of 23S rRNA that binds them (Proux *et al*., 2011). Besides SrmB, several related helicases are present in *E. coli*: DeaD (Jain, 2018), DbpA [RhlC, (Lopez de Victoria *et al*., 2017)], RhlB [also MmrA, (Bruce *et al*., 2018)] and RhlE (Cartier *et al*., 2010). The two‐first ones are involved in ribosome biogenesis while the two latter are involved in RNA degradation. A helicase function of this family is essential for ribosome biogenesis not only in model bacteria such as *E. coli* and *B. subtilis*, where several members of the family are present simultaneously, but at least as a specimen of one of these proteins in reduced genomes such as that of *B. aphidicola* or *M. mycoides* (Table 1).

In parallel, there is overlap between the ATP‐dependent helicase family used in discrimination of active structures during ribosome biogenesis and degradation of misfolded ribosomal RNA or messenger RNA turnover. This is witnessed by the observation that double‐deletion strains lacking ribonuclease R (RNase R) and either the DeaD or SrmB helicases display growth defects and an enhanced accumulation of ribosomal RNA fragments (Jain, 2018). RNase R is a bifunctional 3′ to 5′ exoribonuclease/ATP‐dependent helicase involved in ribosome quality control when associated with RnrY(YbeY) (Chu *et al*., 2017). Its helicase activity combines two ATP‐binding regions, with the Walker A motif located in the C‐terminal region of the protein, while the Walker B motif is in its N‐terminal region. This organization implies that the two parts of the protein must come together to generate a functional ATP‐binding site. Remarkably, ATP binds only when double‐stranded RNA is present. Furthermore, the nuclease activity of RNase R is not needed for its helicase activity. Again, as in the case of other MxDs, ATP hydrolysis is not required for the informational discrimination step since both ATP‐gamma‐S and AMP‐PNP stimulate helicase activity, as can other nucleoside triphosphates (Hossain *et al*., 2015; Heller *et al*., 2017), but ATP hydrolysis allows the enzyme to start a new round of activity.

Further discrimination processes involve ATP‐dependent helicases. The omnipresent degradosome complexes [that differ in different clades (Danchin, 2009a)] comprise widespread helicase RhlB or its equivalent in interaction with polynucleotide phosphorylase (Tseng *et al*., 2015). By contrast, highly specific discriminating RNA helicases were selected in different organisms, presumably to cope for specific functions related to the ecological niche of the organism. In this context, we identified in Enterobacteria (and sometimes in particular species or strains) several such enzymes (Table 1). HrpA is processing specific mRNAs involved in fimbrial operons possibly involved in adhesion to host cells. It is present in fairly small genomes such as that of *Akkermansia muciniphila* or Spirochetes (Salman‐Dilgimen *et al*., 2013). In the same way, HrpB(YadO) is an ATP‐dependent RNA helicase with highly specific function in the formation of pili (Pietrzyk‐Brzezinska *et al*., 2018), probably via modulation of specific mRNAs or regulatory RNAs half‐life.

### Managing maintenance

Life is closely tied to the physics of soft matter. Standard management of the corresponding chemistry will let any object, when free of constraints other than temperature, occupy as many available space and energy states as possible, with a probability distribution depending on the temperature. For complicated objects, this implies often the need to pass through contorted shapes or cross barriers of high levels of activation energy. Protein folding is a critical case in point as mismanagement of folding results in non‐functional or even toxic proteins. During protein synthesis, folding is operated in an algorithmic way, from the amino‐terminus to the carboxy‐terminus, via interaction with the ribosome as a first folding chaperone. Subsequently, trigger factor (Tig), an omnipresent chaperone located at the tunnel where the polypeptide in statu nascendi exits the ribosome, channels folding along a path that has been the result of a very long process of natural selection. Tig has to recognize the nascent polypeptide but does not need to discriminate its features against similar features (Wruck *et al*., 2018). Hence, it is not energy‐dependent, at least directly. As a consequence, this factor is not absolutely essential, and mutants devoid of the protein can survive. To be sure (see Table 1), present in *M. mycoides* Syn1.0, it has been deleted from *M. mycoides* Syn3.0, where it is possibly replaced by another putative chaperone (Danchin and Fang, 2016).

By contrast, other chaperones that act on completed proteins are strictly energy‐dependent. Their role is to discriminate between functional and non‐functional proteins, preventing spurious self‐aggregation, and possibly restoring the latter to their functional state or, sometimes, rerouting them to a degradation system, as non‐functional proteins must often be disposed of. Specific energy‐dependent proteases have been selected to this aim. We summarize how both of these processes develop, discuss the energetics of all these processes and then briefly conclude with the question of the way cell's cope with this inevitable fate of all things.

#### Energy‐dependent molecular chaperones

Cells harbour several types of energy‐dependent molecular chaperones. Three major types that act in synergy are (almost) always simultaneously present: chaperonin GroES/GroEL (Yan *et al*., 2018), molecular chaperone DnaK/DnaJ/GrpE (Uchida and Kanemori, 2018) and disaggregase ClpB (Sugita *et al*., 2018). As seen in the minimal genomes of *B. aphidicola* or *M. mycoides* Syn3.0 (Schwarz *et al*., 2018), the presence of at least two of them is essential for life. When cells require iron–sulfur centres, a further set of specific chaperones, HscAB, may also be required (Table 1). Refolding proteins could be purely entropy‐driven, via the screening of water molecules by a molecular chaperone at surface hydrophobic residues of the protein of interest [see the rationale of using hydrophobic solvents in (Dreyfus *et al*., 1978)]. However, more often than not, refolding of misfolded polypeptides is likely to also involve a mechanical step (obviously this would require energy) coupled to information management meant to identify those proteins that need to be disaggregated, refolded or degraded. This implies that more than one energy‐dependent step will be involved in the process and that the presence of other partners is often required. As a case in point, the common chaperone HtpG(Hsp90) interacts with DnaK(Hsp70) to remodel heat‐inactivated proteins. As with other MxDs, HtpG undergoes substantial nucleotide‐dependent conformational rearrangements. It binds RpoH, and gene *htpG* is in the *rpoH* regulon. The regular functional collaboration between HtpG and DnaK requires that the two chaperones directly interact (Genest *et al*., 2018). Within the frame of the present work, we can predict that inhibiting ATPase activity would prevent recycling of the protein to its ground state and abolish its activity. In contrast, we could also predict that enhancing ATPase activity unspecifically would affect target recognition, resulting in high toxicity. This is exactly what is observed when cells are submitted to the action of the compound zerumbone that modifies cysteine residues of cyanobacterial HtpG (Nakamoto *et al*., 2018).

Almost ubiquitous, GroES/EL drives the final step of information transfer in the expression of many genes, the step of chaperonin‐mediated protein folding. Curiously, while investigators were quite aware of an involvement of information at this stage – the expression ‘proof‐reading’ is commonplace in articles about this chaperonin, they did not care to identify the way energy was involved in creating or recruiting information *per se*. GroES/EL is a protein complex with sevenfold symmetry, a feature presumably meant to allow the complex to freely explore the cytoplasm, because it cannot form large ordered structures (Mikhael *et al*., 2010). A set of seven GroEL subunits binds 7 ATP molecules, then associates to a lid made of seven GroES subunits and further associates with itself by mirror symmetry. Among the ATP molecules bound to the GroEL heptamer, two or more are necessary (and sufficient) to drive the binding of GroES co‐chaperonin to the GroEL complex. Subsequently, binding of 4 or more ATP molecules is needed for any degree of productive substrate protein folding, in particular for driving the bound protein out of the GroEL cage into the GroES‐encapsulated folding cavity (Chapman *et al*., 2008). In works spanning three decades, Goloubinoff and co‐workers explored the pathway of ATP metabolism in the GroES/EL complex and observed that it required two steps. In the first step, which does not imply ATP hydrolysis, the chaperones spontaneously recognize, bind and prevent the aggregation of denatured polypeptides. At this stage, ATP, but not ATP hydrolysis, is required to recruit the GroES subunit to the active complex, discriminating relevant substrates against native proteins. In the second step, that requires ATP hydrolysis, the bound denatured polypeptides are mechanically dissociated in a controlled manner such that they may properly refold and assemble into native oligomers (Finka *et al*., 2016). This sequence of events fits remarkably well with the description of a MxD: first, binding to misfolded protein, in the presence of ATP, then dissociation and ATP hydrolysis, which resets the initial state of the unfoldase.

The outcome of this process results in splitting the set of proteins into two classes, those which are misfolded (and then refolded or discarded) and those which are properly folded. Energy is used to place the unfoldase into a conformation prone to bind to a specific class that of misfolded proteins. In this process, the presence of ATP may be required for the initial step, which leads to proper folding in a sequence of out of the equilibrium steps (Goloubinoff *et al*., 2018). Interestingly, the unfolding step does not necessarily require ATP hydrolysis (i.e. does not always require mechanical energy), as illustrated with the behaviour of the Tig chaperone for example, or by the fact that unfolded proteins may fold back properly if they are given enough time to do so. This feature has indeed been observed with RuBisCO (Viitanen *et al*., 1990). This demonstrates that the energy‐dependent mechanical part of the folding process is not the key role of energy dissipation in (re)folding proteins. By contrast, this highlights the essential role of energy in information management.

Another way of managing information, also requiring energy for resetting the system to its ground state, is illustrated by two chaperones, DnaK, associated with its co‐chaperone DnaJ and nucleotide recycling factor GrpE, and ClpB, which can be solely used for protein refolding or for delivery of misfolded or erroneous proteins to a degradation system. DnaK belongs to the family of Hsp70 ‘heat‐shock’ proteins. It does not form cage‐like structures and can therefore accommodate large substrates. It acts as a disaggregase in addition to its unfoldase activity (Glover and Lindquist, 1998). The sequence of disaggregating events involves co‐chaperone DnaJ that drives the DnaJ/DnaK complex to target structures (short hydrophobic patches in misfolded or aggregated substrates), in an ATP‐dependent process, followed by ATP hydrolysis and recycling of the complex with ADP release upon binding of GrpE (Mayer and Gierasch, 2018). Again this process, similar to processes involving G‐proteins, follows Landauer's principle with a typical MxD behaviour.

ClpB is another omnipresent unfoldase (Table 1) that translocates polypeptides through its axial channel. ClpB is non‐processive, catalysing protein complexes disaggregation by taking one or two translocation steps followed by rapid dissociation (Li *et al*., 2015). It can act alone or in association with DnaK. ClpB and DnaK cooperate (with DnaJ and GrpE) in the bichaperone‐mediated, ATP‐dependent unfolding of protein aggregates (Fernandez‐Higuero *et al*., 2018). ClpB must distinguish between properly folded and aggregated proteins by recognizing specific physical and/or chemical surface properties of the aggregates. In *E. coli,* ClpB exists in two isoforms, long ClpB(alpha) and short ClpB(beta), which is initiated from an internal GUG start at codon 149. The N‐terminal domain, present only in the ClpB long form, is required for efficient binding to protein aggregates and fully effective chaperone function. Coordinated expression of both long and short forms of ClpB is required for the protection from thermal killing (Ranaweera *et al*., 2018; Sugita *et al*., 2018). As observed repeatedly in the factors described in our previous discussion, ClpB has two ATP‐specific sites, and ATP binding is triggering multimerization. Binding to both sites is important, in an asymmetrical way, for formation of an hexamer of the protein. As in the case of the GroESL chaperonin, ATP hydrolysis is split into several stages, with the hydrolysis of 1–2 ATP molecules per hexamer inducing a cycle of structural changes. Binding of ATP prior to hydrolysis results in the formation of a ‘tense’ hexameric structure (Uchihashi *et al*., 2018), ready to open up and bind its target substrate. That the major role of ATP binding and hydrolysis is informational is reflected by the observation that ClpB can passively – i.e. without energy dissipation – thread soluble denatured proteins through its central channel (Nakazaki and Watanabe, 2014). Overall, we have here a situation matching that of chaperonins, but with a distinct sixfold symmetry. Structural transitions of the hexameric ClpB ring match well the ratchet‐like polypeptide translocation mechanism proposed for the ClpB eukaryotic counterpart Hsp104 (Gates *et al*., 2017). A further role of ClpB is to funnel misfolded proteins into a degradation system, as we now see.

#### Energy‐dependent substrate‐specific proteases

‘Housecleaning’ is a necessity in any sustainable closed cell metabolism. With ClpB and other unfoldases, we have seen how disaggregation plays an essential role. Yet, once aggregates are split into parts, it is likely that some of them will not re‐establish any functional activity. The cell must then get rid of them. In the case of proteins, this critical function is performed by proteases. However, unless highly specific, such as enzyme RppA that cleaves off the first nine residues of protein L27 in Firmicutes and Tenericutes (Danchin and Fang, 2016), proteases cannot be synthesized in the cytoplasm, as active forms of these enzymes would wipe out the main components of the cell. Yet, proteolysis of wrong proteins is of crucial importance as they would interfere with the normal life of the cell. They may even sometimes catalyse the spontaneous propagation of their flawed form, as witnessed in the case of prion‐like proteins (Chakravarty and Jarosz, 2018).

To prevent this negative outcome, there exist cytoplasmic proteases. However, these enzymes are energy‐dependent. This presents a paradox, because proteases are expected to be exothermic and certainly not to consume ATP (Danchin *et al*., 2011). Be that as it may, following the arguments developed previously, we expect that the proteases meant to discriminate wrong proteins from functional ones would be energy‐dependent, acting as MxDs do. Discrimination of the protein substrates to be degraded could be direct or the effect of an accessory factor. As a case in point, this fits with the fact that ClpB funnels its defective substrates for degradation into multipurpose protease ClpP. This protease breaks down specific proteins recognized by other unfoldases such as ATP‐dependent unfoldase ClpX [that has a single ATP‐binding site per subunit (LaBreck *et al*., 2017)], or members of the ClpA family [that have two ATP‐binding sites per monomer (Miller *et al*., 2018), as do the majority of the proteins we considered here]. An important role of the proteases is degradation of the SsrA‐tagged incomplete proteins generated when translation has been accidentally interrupted. The interaction between ClpX and ClpP is still enigmatic, as ClpX is an hexameric protein, while ClpP is an heptamer. This creates an intrinsic asymmetry in the behaviour of the complex (Fux *et al*., 2018). Despite extensive study, complete understanding of how the nucleotide‐binding domains coordinate ATP binding and hydrolysis to polypeptide translocation is currently lacking. Again, the role of ClpX or ClpA has been interpreted as purely mechanical (Bell *et al*., 2018), but we can be confident that, besides a likely mechanical action required to unfold their substrates, these proteins must also manage information, as previously discussed. A MxD activity via energy‐dependent selection of targets should be explored in priority.

In the same way, another ubiquitous protease is required for cleaning up defective regulators in a large number of bacteria (including in *B. aphidicola*, Table 1). This is the case of protease ClpQ(HslV) associated with its ATP‐dependent specificity factor ClpY(HslU) that degrades SulA, RcsA, RpoH and TraJ as well as RNase R (Tsai *et al*., 2017). Also, cleaning up defective membrane or periplasmic proteins is realized by ATP‐dependent protease FtsH, which behaves as ClpP does (Ruer *et al*., 2018), acting in concert with factors HflC and HflK in *E. coli* (but not in Firmicutes).

Finally, there are conditions when cells cannot sustain a significant level of ATP, for example during stationary phase survival. Yet it is important that these cells also cope with abnormal proteins or aggregates. Under such conditions, the ultimate energy reserve is polyphosphate (polyP), an omnipresent mineral that is insensitive to harsh physical conditions. PolyP can substitute for ATP in many important reactions [see (Danchin, 2009d) for review]. In particular, it can be used by a multipurpose energy‐dependent protease, Lon (protease Ia), that has an essential role when cells age and is ubiquitously present, sometimes in multiple copies (Table 1). Lon substrate specificity has been explored early on (Perez‐Martin and de Lorenzo, 1996), but the list of its targets keeps increasing. Besides known *E. coli* Lon targets, namely RecA, RuvB and IbpA, this protease copes with processes linked to all kinds of stresses, including ageing, sulfur metabolism, the superoxide stress response, nucleotide biosynthesis, amino acid and central energy metabolism (Arends *et al*., 2018). The coupling between ATP hydrolysis and proteolysis has been extensively studied in this protein. This protease degrades various proteins to fragments of 5–20 amino acid residues. In early studies, it was observed that the enzyme hydrolysed about two molecules of ATP for each new fragment, identified by its amino‐terminal group (Menon *et al*., 1987). More recently, the picture of proteolytic processing was described in details, with identification of several steps including recognition of the proper substrate, and unfolding and degradation. An important feature of Lon catalysis is that ATPase activity is not directly parallel to proteolytic cleavage, demonstrating that ATP has a further role in addition to assisting proteolysis. In fact it was observed the non‐hydrolysable analogue of ATP, AMP‐PNP is enough to trigger proteolysis, while ATP hydrolysis increases the overall speed of the reaction (Mikita *et al*., 2013; Brown *et al*., 2018), a feature that is exactly as expected for the behaviour of a MxD, where the recycling step would require an energy‐dependent reset. It would be important to explore whether the other MxDs discussed here can also accommodate polyP as an energy source.

## Cells and computers: informational versus mechanical energy

Hitherto, we have explored the role of nucleoside triphosphate‐dependent hydrolysis as an energy source for MxDs. When reaching this point of the present work, persistent readers may wonder why it took biologists so long to recognize that cells had to implement, besides DNA replication, an energy‐dependent specific way to create and keep information of the DNA ‘reading machine’ – the chassis of synthetic biology specialists – for the future of their progeny. To account for this paradox they may note that, after all, ATP hydrolysis would entail an energy dissipation of a much larger order of magnitude than Landauer's limit, *kT*ln2. To be sure, in *E. coli* cells growing on glucose, a value of ≈20 *kT* was reported for average ATP hydrolysis (Tran and Unden, 1998). Thus, to place things in perspective, erasing one bit of information requires about 3% of the hydrolysis of one ATP molecule into ADP, an information of about 30 bits. Does this reject our line of reasoning? We contend that, rather than an objection, understanding this discrepancy reveals that cells – that operate at temperatures of the order of 300 K – are immensely better energy‐efficient computing devices than are our computer artefacts. Yet, nobody would challenge the fact that the latter devices are meant to manage information.

Life operates far from absolute zero temperature, in a region where thermal noise is not insignificant for biochemical processes. Now, Landauer's limit is of the same order of magnitude as thermal noise, also in the same range as the energy of one hydrogen bond. This implies that any concrete process that expects to create information within cells – to allow them to compute – has to find a way to increase significantly the corresponding signal‐to‐noise ratio. This summons the involvement of a specific ‘amplifier’ that ties up the dissipation of Landauer's limit energy to a much higher energy‐dissipative process. Remarkably, constructing such an amplifier with atoms fits with the challenge of implementing an abstract information‐managing process (resetting a memory) into the physical world made of compounds endowed with mass and energy, and developing in space and time. A MxD is an abstract massless entity. Yet, its physical implementation requires that it is made of concrete atoms. Worse, it also has to perform its actions, which, in the initial massless metaphor, does not entail mechanical energy dissipation. By contrast, once made of atoms, mechanical constraints ask for a counterpart that dissipates some energy. The movements of an authentic MxD nanomachine are mechanical, and this cannot be free from energy dissipation. As a consequence, when we say that the demon must be reset to its original state, this implies both an informational reset and a mechanical reset.

This is the price to pay to implement an abstract requirement into the world of mass and energy. The consequence is that, if information management is indeed a critical feature, it must be associated with a concrete entity that will use energy well above the constraints of noise. We propose that this is exactly the raison d’être of the use of one ATP molecule in the material implementation of a concrete MxD. The living MxD used an amplification of the basic energy requirement of Landauer's principle, making ATP hydrolysis the basic ‘quantum’ of energy necessary to manipulate information, instead of the abstract physical requirement, which is much lower. In mechanical terms, hydrolysis of one ATP molecule is twice the energy necessary to put into motion a molecular motor that exerts a force of roughly 5 pN over a 10 nm step size, producing work of the order of 50 pN nm (Phillips *et al*., 2012). This is sufficient for the mechanical actions associated with the MxD while it operates its discriminative action. Briefly, the cost of creating a material embodiment of a MxD of the order of 30 times the Landauer's limit is particularly low‐priced. We think that this apparent discrepancy, necessary to place the action of the MxD well above thermal noise, is the reason why the energy cost involved in management of information in cells has been overlooked.

*Is this view far‐fetched?* To place the argument in perspective, with cells pictured as computers making computers (Danchin, 2009e), we might like to evaluate how our authentic computers fare. Indeed, computers are also material devices that need to use massive objects to manipulate information. How far are they from the figures that can be computed from Landauer's principle? Comparison with cells is highly telling. The point is that energy efficiency of real contemporary computers is vastly lower than that of living cells. The cost of an elementary computer's operation is of the order of 25 nanojoule (Sandberg, 2016), while Landauer's limit at 20°C is of the order of 2.75 zeptojoule! Of course, the energy cost of computers halves by a factor of 1.57 every year (Koomey *et al*., 2011), but we are, and will remain for a long time, very, very far from the remarkable efficiency of cellular computation.

Finally, the origin of MxDs asks highly relevant evolutionary questions. In many of the examples provided here, we observed that MxDs comprised structures with two nucleoside triphosphate‐binding sites, most often two ATP sites, but sometimes one GTP and one ATP site or even two GTP sites. Exploring the function of enzymes that use two triphosphates (nucleotide kinases or cyclic dinucleotide cyclases for example) should be explored in priority. The latter might be particularly revealing as they couple signal recognition to their cyclase activity. Evolution of these proteins might have led to functions that use an extra or apparently inessential nucleotide in the elusive function of MxDs.

## Conclusion

The cell is the atom of life. Constructing living factories requires us to understand the way it dissipates energy. Among its many original features, the cell has to distinguish whether something belongs to it or is of foreign nature. Understanding how this proceeds is key for the future developments of synthetic biology. There is no life without an implementation of a process allowing creation and continuity of an identity. Since the time of the famous book of Erwin Schrödinger *What is Life?* most life scientists have explored the thermodynamic behaviour of living cells in terms of entropy control, in general perceived as a fight against disorder. In most approaches, this view was linked to information only via the similarity of the mathematical description of entropy in large systems of equivalent entities and that of information carried by a message as described by Claude Shannon. Briefly, behaviours that could be described in a Σ *p*_*i*_ ln*p*_*i*_ law were implicitly assumed to result from a common cause, pertaining to concepts that remained fuzzy, such as order or information. Yet, the very nature of information has long been explored with much deeper concepts, such as algorithmic complexity (Kolmogorov, 1963) or logical depth (Bennett, 1988b). These modern views progressively witnessed a revival of the thought experiment proposed by Maxwell almost 150 years ago, where a process involving an action following the measure of a particular state had to be reset in order to keep performing it in a recurrent manner (Leff and Rex, 2002).

A limited number of life scientists have perceived the importance of this view [see in particular (Mommaerts, 1966), where the very idea of authentic MxDs acting in cells is deemed irrealistic or even impossible, however]. In life sciences, previous literature on MxDs comprises only a handful of papers [e.g. (Walker, 1976; Wang and Wang, 1979; de Meis *et al*., 1992; Donachie, 2002; Balbin and Andrade, 2004; Gatenby and Frieden, 2016; Nath, 2016)], sometimes with an explicit link to management of information (Adami *et al*., 2000). Within this literature, a few articles linked MxDs to creation of information with emphasis on the importance of memory reset (Danchin, 1996, 2009c, 2012; Binder and Danchin, 2011; de Lorenzo *et al*., 2014; Mehta *et al*., 2016; Wen, 2018), an essential feature of the MxD identified by Landauer and Bennett (Landauer, 1961; Bennett, 1988b; Danchin, 1996). Using a general analysis of a set of functions that appear to be omnipresent in cells, we have tried here to summarize this reflection, while identifying critical functions where information management is key. Interestingly, the quest of a minimal set of functions meant to design a well‐functioning synthetic biology chassis can help us identify the minimal presence of MxDs in all living cells. This work is an effort in this direction, and there is no doubt that we must have overlooked some of the functions that should be attributed to MxDs. It is remarkable that about 50 MxD‐coding genes, within a minimum of < 500 needed for life to develop, are required to allow the development of an autonomous cell. Furthermore, we have restricted our quest to processes involving nucleoside triphosphate hydrolysis. Other energy sources, such as the omnipresent methylation processes, might also deserve investigation. Finally, exploring how cells cope with ageing will help us uncover further roles of Maxwell's demons.

## Conflict of interest

None declared.
